# Comparative Quality Evaluation of Physicochemical, Technological, and Protein Profiling of Wheat, Rye, and Barley Cereals

**DOI:** 10.3389/fnut.2021.694679

**Published:** 2021-09-16

**Authors:** Monika Rani, Gagandeep Singh, Raashid Ahmad Siddiqi, Balmeet Singh Gill, Dalbir Singh Sogi, Mohd Akbar Bhat

**Affiliations:** ^1^Department of Food Science and Technology, Guru Nanak Dev University, Amritsar, India; ^2^Department of Chemistry, Guru Nanak Dev University, Amritsar, India; ^3^Dean McGee Eye Institute Oklahoma University of Health Sciences Center, Oklahoma City, OK, United States

**Keywords:** wheat, rye, barley, solvent retention capacity, SDS-PAGE, amino acids

## Abstract

Agronomically important cereal crops wheat, barley, and rye of the Triticeace tribe under the genus Triticum were studied with special focus on their physical, proximal, and technological characteristics which are linked to their end product utilization. The physiochemical parameters showed variability among the three cereal grains. Lactic acid-solvent retention capacity (SRC) was found to be higher in wheat (95.86–111.92%) as compared to rye (53.78–67.97%) and barley (50.24–67.12%) cultivars, indicating higher gluten strength. Sucrose-SRC and sodium carbonate-SRC were higher in rye as compared to wheat and barley flours. The essential amino acid proportion in barley and rye cultivars was higher as compared to wheat cultivars. Barley and rye flours exhibited higher biological value (BV) owing to their higher lysine content. SDS-PAGE of wheat cultivars showed a high degree of polymorphism in the low molecular range of 27.03–45.24 kDa as compared to barley and rye cultivars. High molecular weight (HMW) proteins varied from 68.38 to 119.66 kDa (4–5 subunits) in wheat, 82.33 to 117.78 kDa (4 subunits) in rye, and 73.08 to 108.57 kDa (2–4 subunits) in barley. The comparative evaluation of barley and rye with wheat cultivars would help in the development of healthy food products.

## Introduction

Wheat is the most utilized cereal for human consumption as compared to rye and barley. Clinical studies have shown that barley and rye play a significant role in reducing the risk of cardiovascular disease (CVD), lower postprandial body glycemic index, improve insulin responses in diabetics, lower serum cholesterol level, protect against obesity, and safeguard against the hormone-related risk of colon cancers (1, 2). The U.S. Food and Drug Administration (FDA) has declared barley as a functional food while the European Food Safety Authority (EFSA) has approved the health claim of rye for maintaining bowel function (3).

The rye grain contains a high amount of total dietary fiber, i.e., 19.9% of dry matter (including soluble dietary fiber, SDF, and insoluble-dietary fiber, IDF) as compared to 15.2% in barley and 13.5% in wheat grains (1). SDFs such as arabinoxylan and β-glucan cause an increase in the viscosity of intestinal contents, regulating slower absorption of sterol and glucose and ultimately maintaining glucose, cholesterol, and insulin levels. Similarly, IDFs like cellulose, lignin, and hemicelluloses owing to their high-water absorption ability increases the fecal volume which prevents short as well as long-term disease complications.

Barley and rye flour have been utilized for the production of different commercial food preparations such as breakfast cereals, barley flour tortillas, soft whole-grain rye bread, crisp bread, soups, porridge, and baby foods, etc. About 65% of wheat production is utilized as human food while its remaining 20 and 15% are used for animal feed and miscellaneous purposes, respectively (4, 5). In barley, about 68% is used for feed purposes, 24% for distillery and brewing industries, 6% for human food, and the remaining 2% for other purposes like bio-fuel generation (6). Similarly, in rye, about 42% is used for livestock feed, 31% for human food mainly as rye bread or other processed products, and 27% for other purposes (2). The primary reason for the disparity in the usage of these cereals for human consumption is apparently due to the remarkable visco-elastic nature of wheat dough. This is one of the reasons why wheat is used in conjunction with many rye and barley products for achieving desired dough properties. Therefore, a comparative investigation on wheat, rye, and barley cultivars with special focus on technological, functional, and physicochemical properties is needed. Hamdani et al. (7) has compared the physical properties of barley and oats cultivars and reported significant differences in physical parameters between cultivars and also among cereals. Rodehutscord et al. (8) studied the physiochemical characteristics and amino acid composition of various cereal grains (barley, rye, maize, triticale, oats, and wheat) and reported substantial differences in their physical, chemical, and amino acid composition. Similarly, Kowieska et al. (9) demonstrated significant variation in crude protein, crude fiber, mineral, and amino acid composition in Polish wheat, rye, triticale, and barley cultivars. Drakos et al. (10) compared the nutritional and functional components of rye and barley flour with reference to milling characteristics. Similarly, few authors have also studied the impact of the addition of barley and rye flour on wheat bread quality (11, 12).

This study has been designed to focus on the physicochemical investigation of wheat, rye, and barley cereals using a multi-technique approach. Technological properties of cereal flours have been evaluated using solvent retention capacity (SRC), SDS-sedimentation volume, water-holding capacity (WHC), and oil-holding capacity (OHC). Further, these studies were extended to understand the gluten characteristics of these flours *via* analysis of wet gluten, dry gluten, WHC, and OHC of gluten. The protein profiling of flours has been investigated using SDS-PAGE and amino acid analysis (AAA) of rye and barley in comparison to wheat. The correlation between different technological and functional component parameters has been derived and discussed in detail. The inferences obtained from this study would help in establishing the relative understanding of the technological properties of cereals under investigation. Therefore, the analytical insights gained would facilitate new product applications pertaining to rye and barley. The obtained information would further promote the end-use application of these grain flours to millers, breeders, and manufacturers for commercial application.

## Materials and Methods

### Raw Materials

The authentic cereal grains of rye cultivars (MCTLG-1, MCTLG-2, MCTLG-3, MCTLG-4, and MCTLG-5) and wheat cultivars (HPW-42, HPW-147, HPW-155, HPW-236, HPW-249, and HPW-349) were procured from Chaudhary Sarwan Kumar Himachal Pradesh Krishi Vishvavidyalaya (CSK HPKV), Palampur, India, located at a latitude of 32° 6′ 52" N longitude 76° 33′ 24" E, and altitude 1,614 m above sea level. Palampur (HP) has a humid subtropical climate (warm summer and cold winters) and sandy to loamy textured soil. The barley cultivars (BH-393, BH-902, BH-946, and BH-959) were procured from Chaudhary Charan Singh Haryana Agricultural University (CCS HAU), Hisar, (latitude 29° 8′ 57.08" N, longitude 75° 43′ 17.95" E and an altitude of 212.78 m above sea level) India. The climate of Hisar is tropical monsoonal (very hot summers, relatively cold winters) with fertile alluvial soil. All the cereal grains were grown during the crop years 2014-15. Brabender Quadrumat junior mill (Brabender OHG, Germany) was used to mill the conditioned grains to obtained flour with an extraction rate of 72, 68, and 60% for wheat, rye, and barley, respectively. It was stored at −20°C and thawed before analysis (25°C for 2 h). All the chemicals used were of analytical grade.

### Methods

#### Grain Characteristics

The grain length (L), width (W), and thickness (T) were measured using a digital vernier caliper (Thermo Fischer Scientific, Waltham, Massachusetts, USA) with an accuracy of 0.01 mm. Geometric parameters such as equivalent diameter (D_m_), L/W ratio, sphericity (Φ), aspect ratio (R_a_), seed volume (V), and surface area (A) were calculated using formulas given in the literature (7, 13, 14) while gravimetric characteristics like thousand kernel weight (TKW), hundred kernel volume, bulk density (D_B_), true density (D_T_), and porosity (ε) were determined by following the procedure of Wani et al. (14).


(1) $$\text{Equivalent diameter }\left({\text{D}}_{\text{m}}\right)={\left(\text{LWT}\right)}^{1/3}$$

(2) $$\text{Sphericity}(\Phi )=\frac{{\left(\text{LWT}\right)}^{1/3}}{\text{L}}\text{X }100$$

(3) $$\text{Aspect ratio }\left({\text{R}}_{\text{a}}\right)=\frac{\text{W}}{\text{L}}$$

(4) $$\begin{array}{l} \text{Seed volume }\left(\text{V}\right)\;=\frac{\;\pi {\text{B}}^{2}{\text{L}}^{2}}{6\left(2\text{L}-3\right)} \end{array}$$

(5) $$\begin{array}{l} \text{where B}={\left(\text{WT}\right)}^{1/2} \end{array}$$

(6) $$\begin{array}{l} \text{Surface area }\left(\text{S}\right)=\frac{\;\pi \text{B}{\text{L}}^{2}}{\left(2\text{L}-\text{B}\right)} \end{array}$$

where, W = width; L = length; T = thickness of grain


(7) $$\begin{array}{l} \text{Bulk density }=\frac{\text{sample weight}}{\text{volume}} \end{array}$$

True density (g/mL) was measured by the liquid displacement method using toluene as a displacement liquid.


(8) $$\begin{array}{l} \text{True density }=\frac{\text{sample weight}}{\left(\text{V}2-\text{V}1\right)} \end{array}$$

where, V1 = initial volume and V2 = final volume


(9) $$\begin{array}{l} \text{Porosity }\left(\varepsilon \right)=100\left[1-\left({\text{D}}_{\text{B}}/{\text{D}}_{\text{T}}\right)\right] \end{array}$$

where ε is the porosity in percentage; D_B_ is bulk density in g/mL, and D_T_ is seed density in g/mL.


(10) $$\begin{array}{l} \text{Hundred kernel volume}=\text{Total volume}-20\text{ mL} \end{array}$$

#### Flour Characteristics

##### Proximate Composition

Moisture (44-15.02), protein (46-12.01), ash (08-02.01), fiber (32-10.01), and fat (30-25.01) were estimated by following the AACC approved methods (15). Carbohydrate content was determined by the difference method, whereas energy values were calculated by multiplying protein and carbohydrate content by 4 kcal/g and fat content by 9 kcal/g (16).

##### Solvent Retention Capacity

Solvent retention capacity (SRC) was determined following the standard procedure as per AACC approved method 56-11 (15). One gram of flour was suspended individually in 5 ml of standard solutions (deionized distilled water, 5% sodium carbonate, 5% lactic acid, and 50% sucrose). The flour suspension was allowed to hydrate and mixed at 150 rpm for 20 min on a horizontal incubator shaker (LSI-3016R, Daihan Lab Tech Co., Ltd., Namyangju, South Korea) and centrifuged (20 min at 1,100 × g). The supernatant was decanted and kept in an inclined position at a 45° angle for 20 min and then the pellet was weighed. SRC values were estimated using the following equation:


(11) $$\begin{array}{l} \text{SRC }\left(\frac{\text{g}}{100\text{g}}\right)=\;\left(\frac{\text{ Wet pellet }\left(\text{g}\right)}{\text{Flour }\left(\text{g}\right)}-1\;\right)\\ \times \left(\frac{86}{\;100-\text{Flour moisture }\left(\frac{\text{g}}{100\text{g}}\right)}\right)\times 100 \end{array}$$

##### Gluten Performance Index

The gluten performance index (GPI), a good indicator of overall gluten strength, was estimated using the data of SRC (17).


(12) $$\begin{array}{l} \text{GPI}=\frac{\text{Lactic acid SRC}}{\text{Sodium carbonate SRC }+\text{ Sucrose SRC}} \end{array}$$

#### SDS-Sedimentation Value

The SDS-sedimentation value of cereal flour was estimated by following AACC approved method 56–70 (15) in which glutenin protein absorbs water and swells in the presence of SDS-lactic acid reagent. Solution A (2% SDS in distilled water) and solution B (one part lactic acid and eight parts distilled water) were prepared separately. Solution A (10 ml) and solution B (2 ml) were mixed to get an SDS-lactic acid reagent. Six grams of flour and 50 ml of distilled water were transferred into a 100 ml measuring cylinder with a stopper and shaken for 15 s, each time at an interval of 2, 4, and 6 min. Immediately after the final shaking, 50 ml of freshly prepared SDS-lactic acid solution was added and the mixture was allowed to move up and down four times each at an interval of 2, 4, and 6 min for 15 s. Immediately after the final inversion, starting the clock from zero, the flour mixture was allowed to settle in the cylinder for 40 min, and the sedimentation volume was recorded in ml.

### Gluten Content

Wet and dry gluten were analyzed according to AACC approved method 38-10 (15) by a hand washing procedure. The dough was prepared by mixing an adequate amount of water (≈12 ml) with 25 g of flour in a porcelain dish. The round ball of dough was kept in a beaker filled with water for at least 60 min. After the stipulated time, the dough was kneaded gently under running tap water over a 75 mm sieve until milky water turned to colorless water. The obtained sticky or dark mass was then kept in a beaker filled with water for another 60 min without any disturbance. Thereafter the gluten was held between two hands and squeezed hard to remove excess water and rolled to a round mass and weighed to get wet gluten. The wet gluten was then dried in an oven operated at 110°C for 24 h, cooled using a desiccator, and weighed to get dry gluten. The values of wet and dry gluten were calculated using the following equations:


(13) $$\begin{array}{l} \text{Wet gluten }\left(\%\right)=\frac{\text{weight of wet gluten }\left(\text{g}\right)}{\text{weight of the sample }\left(\text{g}\right)}\text{X }100 \end{array}$$

(14) $$\begin{array}{l} \text{Dry gluten }\left(\%\right)=\frac{\text{weight of dry gluten }\left(\text{g}\right)}{\text{weight of the sample }\left(\text{g}\right)}\text{X }100 \end{array}$$

### Color of Cereal Grains/Flour and Dried Gluten Content

The color of the different cereal grains, flour, and gluten was measured using the Hunter Color lab (Hunter Associates Laboratory Inc., Reston USA) by following the procedure of Siddiqi et al. (16). Hue and chroma were determined using the following formulas:


(15) $$\begin{array}{l} \text{Hueangle }\left({\text{H}}^{0}\right)=\text{ta}{\text{n}}^{-1}\left({\text{b}}^{*}/{\text{a}}^{*}\right) \end{array}$$

(16) $$\text{Chroma }(\text{C}*)={\left({\text{a}}^{*2}+{\text{b}}^{*2}\right)}^{0.5}$$

### Amino Acid Analysis of Flour

AAA of flour was performed by following the procedure of Siddiqi et al. (16) with a slight modification. Defatted flour (15 mg) was hydrolyzed using 6N HCl containing 0.1% ß-mercaptoethanol in an autoclave at 110 ± 2°C for 16 h. The digested sample was filtered, evaporated to dryness under a vacuum at 60°C in a rotary evaporator (Buchi, Fawil, Switzerland), re-dissolved with a suitable volume of 0.1 N HCl, and filtered through 0.22 μm filter paper. Amino acid analysis was performed using an amino acid analyzer (Shimadzu, Kyoto, Japan) equipped with pre-column derivatization using three derivatizing reagents such as mercaptopropionic acid, o-phthaladehyde, and 9-fluorenylmethoxycarbonyl chloride. A C-18 column (Acclaim Thermoscientific 120Å, 5 μm, 4.6 × 250 mm; Thermo Fisher Scientific, Waltham, USA) with pH stability of 2–8 was used for chromatographic separation. Analysis was performed by the standard operating manual procedure, using 20 mmol/L of phosphate (potassium) buffers (pH 6.5) as solvent A and 45/40/15 acetonitrile/methanol/water as solvent B. The separation was obtained at a flow rate of 1 ml/min using a gradient elution that allowed 0% of B at 0.01 min, followed by linear raise of eluent B to 50% at 41 min and then again decreasing solvent B to 0% at 44 min at a column oven temperature of 40°C. The injection volume of the standard or sample was 1 μL. The pre-column derivatized amino acids were detected with the help of a fluorescence detector with excitation and emission set at 330 and 450 nm, respectively. Lab solutions LC/GC (Shimadzu, Kyoto, Japan) was used as a working station. The amino acid standard mixture was prepared by mixing 18 amino acids (SRL, Mumbai, India) in 0.1 N HCl which included aspartic acid (Asp), glutamic acid (Glu), serine (Ser), glycine (Gly), threonine (Thr), histidine (His), alanine (Ala), arginine (Arg), tyrosine (Tyr), valine (Val), methionine (Met), cystine (Cys), phenylalanine (Phe), tryptophan (Trp), isoleucine (Ileu), leucine (Leu), lysine (Lys), and proline (Pro). The amino acid comparison of the standard mixture and the digested sample was done based on retention time as well as for the area under the peak for detection and quantification of each eluted amino acid. The estimated glutamic acid and aspartic acid were represented by a combination of acid and amide derivative, as amides such as glutamine (Gln) and asparagine (Asn) are deaminated to glutamic acid (Glu) and aspartic acid (Asp) during acid hydrolysis (18). Therefore, results are expressed as Glu + Gln and Asp and Asn.

### Digestible Indispensable Amino Acid Score

The recommended dietary allowances (RDA) of amino acid for children (age: 6 months to 3 years) was used as the reference protein for the calculation of DIAAS of selected cereals using standard values of leucine (6.6/100 g), lysine (5.7/100 g), phenylalanine (5.2/100 g), valine (4.3/100 g), isoleucine (3.2/100 g), threonine (3.1/100 g), methionine (2.7/100 g), histidine (2/100 g), and tryptophan (0.85/100 g) (19).


(17) $$\begin{array}{l} \text{AAS }\left(\%\right)=\frac{\text{Amino acid in test protein }\left(\text{g}\right)}{\text{Amino acid in the reference protein }\left(\text{g}\right)}\;\times 100 \end{array}$$

### Biological Value

Biological value was calculated using the following equation described by Oser (20).


(18) $$\begin{array}{l} \text{BV}=1.09\;\left(\text{EAAI}\right)-11.73 \end{array}$$

Where EAAI - essential amino acid index, is a percentage of the geometric mean of the ratios of EAA in the test protein relative to their respective amount in the FAO/WHO scoring pattern.

### SDS-PAGE of Total Flour Proteins

Defatted flour (25 mg) was added to 1 ml of 2× Laemmli sample buffer solution (pH 6.8 containing 62.5 mM of Tris–HCl, 25% glycerol, 5% ß-mercaptoethanol, 2% SDS, 0.01% bromophenol blue) in 1.5 ml Eppendorf tubes. The tubes were vortexed to disperse the flour through mixing, horizontal shaking in an orbital shaker at 151 rpm for 1 h at 45°C, heating at 100°C for 5 min in a water bath, and centrifugation (RC 4815S, Eltek, Mumbai, India) at 11,000 × g for 15 min. The supernatant (10 μl) was loaded in each well (Mini-Protean Tetra Cell, Bio-Rad Laboratories, Hercules, USA). Proteins were separated using 4% stacking gel and 12% resolving gel while the current was kept constant at 25 mA until the tracking dye reached the bottom of the gel which was then removed and stained overnight using 0.1% Coomassie Brilliant Blue-R250 in 40% methanol and 10% acetic acid. The gel was destained using 20% methanol and 10% acetic acid. A broad-ranged molecular marker (GeNei, Bangalore, India) was used as a standard consisting of peptides of 205 kDa (myosin), 97.4 kDa (phosphorylase B), 66.0 kDa (bovine serum albumin), 44.0 kDa (ovalbumin), 29.00 kDa (carbonic anhydrase), 20.10 kDa (soybean trypsin inhibitor), 14.30 kDa (lysozyme), 6.50 kDa (aprotinin), and 3.50 kDa (insulin). The quantification of destained gel was analyzed using a Bio-Rad EZ imager (Bio-Rad Laboratories, Hercules, USA). Classification of total prolamin was done according to Schalk et al. (21). SDS-PAGE gels were performed in duplicates.

### Statistical Analysis

The results were expressed as mean ± SD and compared statistically at *p* ≤ 0.05, using one-way analysis of variance (ANOVA) with Tukey's *post-hoc* test performed *via* Minitab software (Version 17, Minitab Inc., State College, PA, USA).

## Results and Discussion

### Physical Characteristics of Cereal Grains

**Figure 1:**
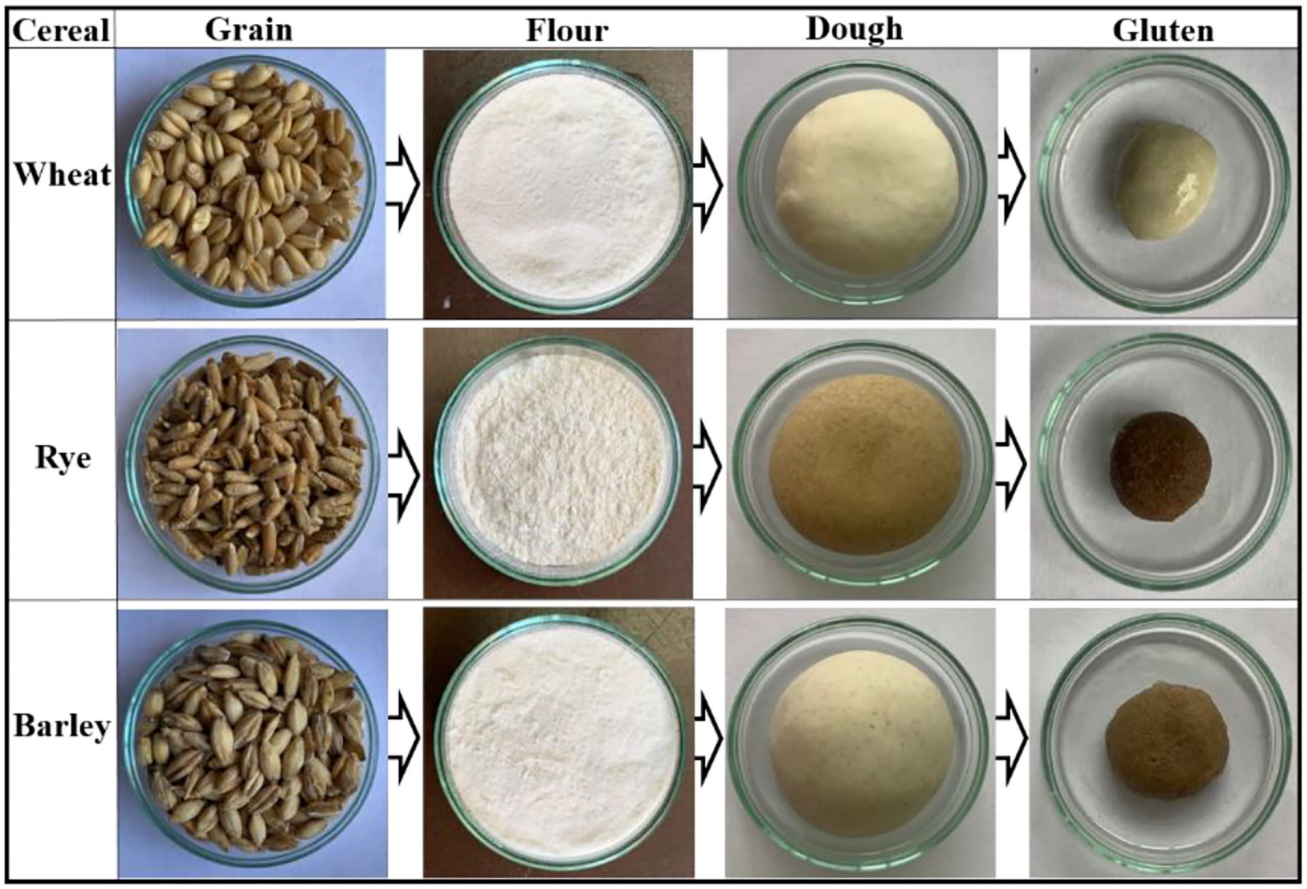
Schematic representation showing the preparation of flour, dough, and gluten from wheat (HPW-142), rye (MCTLG-1), and barley (BH-393) cultivars.

The shape and size are the determinants for the quality evaluation, grain screening, and heat-mass transfer calculations (14). These characteristics are defined by the geometric parameters and are provided in Table 1, Figure 1, and Supplementary Figure 1. The values of length (L), width (W), and thickness (T) of wheat, rye, and barley were found to be in the range 6.62–7.85 mm, 2.25–3.69 mm, and 2.08–3.13 mm, respectively. The grain length of barley (7.06–7.85 mm) and wheat (7.03–7.59 mm, except wheat cultivar, HPW-349, 6.62 mm) cultivars was observed to be slightly higher compared to rye cultivars (6.88–7.45 mm). Similarly, the thickness and width of cereals followed the order of wheat (T, 2.74–3.13 mm; W, 3.41–3.69 mm) > barley (T, 2.42–2.62 mm; W, 3.39–3.55 mm) > rye (T, 2.08–2.35 mm; W, 2.25–2.48 mm) cultivars. The L/W ratio of cereal grains followed the order: rye (2.87–3.07) > barley (2.11–2.31) > wheat (1.85–2.13) cultivars. The high L/W ratio of rye cultivars implied the cylindrical shape of these grains whereas the barley and wheat cultivars had more of an oval morphology of their grains in their respective manner (22). The overall analysis of geometric dimensions revealed that the wheat grains were mostly oval, medium-sized, and wider compared to barley cultivars which were oval, longer, and less wide, while the rye cultivars were relatively cylindrical, shorter, and thinner (Supplementary Figure 1). The sphericity (Φ) and aspect ratio (R_a_) of the cereal grains were found in the range of 45.65–60.81% and 0.33–0.54, respectively. The order of Φ for the cereal grains followed the trend: wheat (56.31–60.81%) > barley (51.04–54.04%) > rye (45.65–47.64%), which implied that the propensity of grains toward rolling out decreased from wheat to rye. Similarly, the R_a_ values had an inverse relationship to the length and the values of cereals followed the trend: wheat (0.47–0.54) > barley (0.43–0.47) > rye (0.33–0.35) which indicated that rye grains were the most elongated and the wheat grains the least. Therefore, a relatively greater magnitude of Φ and R_a_ for wheat and barley cultivars, i.e., highly spherical and less elongated geometry is indicative of the tendency of these grains to roll out as compared to rye, which would prefer to slide over a flat surface (7, 13, 16).

**Table 1:** Geometrical and gravimetrical properties of different wheat, rye, and barley cultivars.

**Cereal**	**Wheat grain**						**Rye grain**					**Barley grain**			
**Cultivar**	**HPW-42**	**HPW-147**	**HPW-155**	**HPW-236**	**HPW-249**	**HPW-349**	**MCTLG-1**	**MCTLG-2**	**MCTLG-3**	**MCTLG-4**	**MCTLG-5**	**BH-393**	**BH-902**	**BH-946**	**BH-959**
L (mm)	7.13 ± 0.18^CDEF^	7.03 ± 0.25^EF^	7.38 ± 0.12^BCDE^	7.59 ± 0.15^AB^	7.04 ± 0.21^EF^	6.62 ± 0.23^EF^	6.88 ± 0.28^FG^	6.94 ± 0.24^FG^	7.11 ± 0.25^DEF^	7.45 ± 0.35^BCD^	7.10 ± 0.20^DEF^	7.53 ± 0.22^AB^	7.50 ± 0.24^ABC^	7.06 ± 0.29^AB^	7.85 ± 0.28^A^
W (mm)	3.61 ± 0.09^AB^	3.65 ± 0.11^A^	3.69 ± 0.07^A^	3.57 ± 0.16^ABC^	3.41 ± 0.07^CD^	3.57 ± 0.08^AB^	2.25 ± 0.08^F^	2.43 ± 0.12^E^	2.44 ± 0.14^E^	2.48 ± 0.11^E^	2.40 ± 0.10^EF^	3.45 ± 0.11^BCD^	3.55 ± 0.12^BCD^	3.39 ± 0.07^D^	3.41 ± 0.10^D^
T (mm)	3.13 ± 0.18^A^	3.07 ± 0.24^A^	3.06 ± 0.18^A^	2.86 ± 0.21^ABC^	2.96 ± 0.19^AB^	2.74 ± 0.29^ABCD^	2.26 ± 0.26^EF^	2.24 ± 0.31^EF^	2.23 ± 0.26^EF^	2.35 ± 0.30^DEF^	2.08 ± 0.26^F^	2.42 ± 0.27^DEF^	2.62 ± 0.34^BCDE^	2.47 ± 0.19^CDEF^	2.52 ± 0.35^CDE^
L/W	1.98 ± 0.06^FGH^	1.92 ± 0.05^GH^	2.00 ± 0.03^FGH^	2.13 ± 0.12^DEF^	2.06 ± 0.06^EFG^	1.85 ± 0.05^H^	3.07 ± 0.19^A^	2.87 ± 0.15^B^	2.91 ± 0.11^AB^	3.01 ± 0.10^AB^	2.97 ± 0.14^AB^	2.18 ± 0.06^CDE^	2.11 ± 0.07^DEF^	2.24 ± 0.08^CD^	2.31 ± 0.10^C^
D_m_ (mm)	4.24 ± 0.10^AB^	4.24 ± 0.11^AB^	4.30 ± 0.09^A^	4.20 ± .0.12^ABC^	4.10 ± 0.08^ABCD^	4.00 ± 0.13^CD^	3.22 ± 0.11^F^	3.31 ± 0.17^EF^	3.34 ± 0.18^EF^	3.46 ± 0.24^E^	3.24 ± 0.16^EF^	3.92 ± 0.18^D^	4.05 ± 0.16B^CD^	3.39 ± 0.15^D^	4.00 ± 0.16^CD^
Φ (%)	59.52 ± 1.35^AB^	60.81 ± 2.08 ^A^	59.29 ± 0.66^AB^	56.31 ± 1.86^BC^	57.56 ± 1.63^AB^	58.73 ± 3.34^AB^	46.90 ± 2.58^E^	47.64 ± 2.29^E^	46.96 ± 1.55^E^	46.46 ± 1.56^E^	45.65 ± 2.08^E^	52.07 ± 1.98^D^	54.04 ± 2.91^CD^	51.84 ± 1.09^D^	51.04 ± 3.45^D^
R_a_	0.51 ± 0.01^BC^	0.52 ± 0.01^AB^	0.50 ± 0.01^BC^	0.47 ± 0.03^DEF^	0.48 ± 0.01C^D^	0.54 ± 0.02^A^	0.33 ± 0.02^H^	0.35 ± 0.02^H^	0.34 ± 0.01^H^	0.33 ± 0.01^H^	0.34 ± 0.02^H^	0.46 ± 0H.01^EFG^	0.47 ± 0.01^DE^	0.45 ± 0.02^FG^	0.43 ± 0.02^G^
V (mm)^3^	26.72 ± 2.01^AB^	26.17 ± 1.88^ABC^	27.35 ± 2.14^A^	25.32 ± 2.54^ABCD^	23.67 ± 1.77^BCDE^	21.93 ± 2.17^DE^	11.66 ± 1.21^F^	12.60 ± 1.92^F^	12.89 ± 2.02^F^	14.31 ± 2.85^F^	11.78 ± 1.84^F^	20.60 ± 2.80^E^	22.73 ± 2.76^CDE^	20.82 ± 2.36^E^	21.72 ± 2.69^E^
S (mm)^2^	49.24 ± 2.58^AB^	48.44 ± 2.36^ABC^	50.38 ± 2.62^A^	48.27 ± 3.06^ABC^	45.39 ± 2.27^BCD^	42.58 ± 2.70^D^	29.04 ± 1.82^E^	30.50 ± 2.92^E^	31.17 ± 3.25^E^	33.73 ± 4.53^E^	29.54 ± 2.79^E^	42.28 ± 3.78^D^	44.92 ± 3.41^BCD^	42.70 ± 3.38^D^	44.25 ± 3.12^CD^
TKW (g)	48.34 ± 0.53^AB^	51.26 ± 0.92^A^	50.53 ± 0.83^A^	50.61 ± 2.08^A^	44.75 ± 0.87^BC^	42.40 ± 1.02^CD^	18.83 ± 0.64^F^	22.32 ± 1.36 ^F^	20.35 ± 0.77 ^F^	22.33 ± 0.80 ^F^	20.60 ± 0.83 ^F^	40.038 ± 3.34^DE^	42.86 ± 2.94^CD^	36.99 ± 2.10^E^	41.99 ± 0.90^CD^
HKV (ml)	5.40 ± 0.89^AB^	5.90 ± 0.42^A^	5.40 ± 0.42^AB^	5.20 ± 0.84^AB^	4.80 ± 0.27^B^	4.80 ± 0.45^B^	2.90 ± 0.22^C^	3.30 ± 0.45^C^	3.30 ± 0.27^C^	3.10 ± 0.55^C^	3.00 ± 0.00^C^	3.00 ± 0.00^C^	3.00 ± 0.00^C^	3.00 ± 0.00^C^	3.10 ± 0.22^C^
D_B_ W/V (g/ml)	0.77 ± 0.02^ABCD^	0.80 ± 0.01^AB^	0.75 ± 0.01B^CDE^	0.78 ± 0.01^ABC^	0.81 ± 0.02^A^	0.79 ± 0.03^AB^	0.74 ± 0.02^CDE^	0.73 ± 0.01^CDE^	0.72 ± 0.01^DE^	0.71 ± 0.01^E^	0.72 ± 0.01^DE^	0.59 ± 0.04^G^	0.63 ± 0.03^FG^	0.61 ± 0.03^FG^	0.64 ± 0.03^F^
D_T_ (g/ml)	1.54 ± 0.03^E^	1.61 ± 0.02^ABC^	1.58 ± 0.01B^CDE^	1.58 ± 0.02^ABCDE^	1.60 ± 0.03^ABCD^	1.61 ± 0.03^AB^	1.57 ± 0.02^CDE^	1.54 ± 0.02^E^	1.56 ± 0.01^DE^	1.55 ± 0.02^E^	1.57 ± 0.01^CDE^	1.61 ± 0.01^AB^	1.61 ± 0.01^AB^	1.58 ± 0.02^ABCDE^	1.62 ± 0.02^A^
ε(%)	49.81 ± 1.27^EF^	50.44 ± 0.95^EF^	52.45 ± 0.36^BCDEF^	50.64 ± 0.93^CDEF^	49.42 ± 1.90^F^	50.66 ± 1.64^DEF^	52.87 ± 1.26^BCDE^	52.60 ± 0.51^BCDE^	53.69 ± 0.57^BCD^	54.17 ± 0.63^B^	53.93 ± 0.76^BC^	63.55 ± 2.66^A^	60.66 ± 1.73^A^	61.63 ± 1.70^A^	60.46 ± 1.84^A^

*L, Length; W, width; T, thickness; L/W, length/width ratio; Dm, equivalent diameter; Φ, sphericity; Ra, aspect ratio; V, seed volume; S, surface area; TKW, thousand kernel weight; HKV, hundred kernel volume; DB, bulk density; DT, true density; ε, porosity*.

*Mean ± SD with different superscripts in a row differ significantly (p ≤ 0.05); n = 5 for each treatment*.

The gravimetric profile of the cereal grains helps in predicting grain soundness, transportation, and storage conditions (23) and is listed in Table 1. In the investigated cereal cultivars, the magnitude of thousand kernel weight (TKW), which indicates the grain quality and expected milling flour yield, followed the order: wheat (42.40–50.61 g) > barley (36.99–42.86 g) > rye (18.83–22.33 g). This suggests that wheat and barley cultivars have greater flour yield compared to rye cultivars. The observed TKW values of cereals exhibited significant differences (*p* ≤ 0.05) both among the cereals and within the cultivars except rye (Table 1). These findings are in line with earlier reports, where TKW values of wheat, barley, and rye cultivars ranged from 45.9 to 52.1 g (24), 40.06 to 41.90 g (7), and 21.6 to 28.5 g (25), respectively.

The bulk density (D_B_) and true density (D_T_) values of the investigated cereal grains ranged from 0.59 to 0.81 g/ml and 1.54 to 1.62 g/ml, respectively. The values of D_B_ observed in wheat (0.75–0.81 g/ml), rye (0.71–0.74 g/ml), and barley (0.59–0.64 g/ml) cultivars suggested that wheat grains were relatively denser as compared to rye and barley. At the intra-cultivar level, wheat cultivar HPW-249 possessed the highest D_B_ value while barley cultivar BH-393 had the lowest. The porosity (ε) values of wheat (49.42–50.66%), rye (52.60–54.17%), and barley (60.46–63.55%) revealed an increasing trend. The porosity (ε) values of cereal grains followed the reverse order of D_B_. The barley cultivar BH-393 (63.55%) exhibited the highest ε value, while wheat cultivar HPW-239 had the lowest.

Among the investigated cereal grains, the wheat cultivars were relatively denser, with low ε, high TKW, and high D_B_ values. High porosity would allow more air flow through the grains which would affect the rate of drying, heating/cooling as well as the amount of energy required to accomplish the process (26) as compared to rye and barley cultivars. The physical parameters (Table 1) of the studied cereals, i.e., wheat, rye, and barley, were near that of the earlier reports (27).

### Colorimetric Analysis

The color characteristics of the cereal grains were evaluated by employing CIE color values (L^*^, a^*^, and b^*^), chroma (C^*^), and hue angle (H^0^). The values of these parameters are provided in Table 2 and Supplementary Figure 1. The lightness of cereal grains (L^*^ value) which is an indication of the light color (0 is black, 100 is white), followed the order: wheat (58.10–63.38) > barley (56.34–61.53) > rye (51.23–53.59). Further, at the intra-cultivar level, wheat cultivar HPW-42 (63.38) was found to possess the highest L^*^ value while rye cultivar MCTLG-5 (51.23) had the lowest. The other color parameters, a^*^ and b^*^ (for a^*^, +ve/–ve = red/green and b^*^, +ve/–ve = yellow/blue), exhibited positive values for all the investigated cereals grains which indicated the presence of a predominantly red and yellow tint in the cereal grains. The magnitude of a^*^ indicated that the extent of red tint decreased in the order: wheat (6.74–7.56) > barley (4.91–5.29) > rye (4.71–4.83). Similarly, the value of b^*^ indicated that the extent of yellow tint decreased in the following order: wheat (18.75–23.62) > barley (16.22–18.71) > rye (13.70–14.76). A relatively greater content of red and yellow tint collectively imparted more brightness to the wheat grains as compared to barley and rye grains. Statistically, the CIE color parameters L^*^, a^*^, and b^*^ were significantly different (*p* ≤ 0.05) for the investigated cereals at inter- and intra-cultivar levels except for a^*^ values where intra-cultivar variation was not significant (*p* ≥ 0.05).

**Table 2:** Hunter color values of grain, flour, and gluten from different wheat, rye, and barley cultivars.

**Cereal**	**Wheat**						**Rye**					**Barley**			
**Cultivar**	**HPW-42**	**HPW-147**	**HPW-155**	**HPW-236**	**HPW-249**	**HPW-349**	**MCTLG-1**	**MCTLG-2**	**MCTLG-3**	**MCTLG-4**	**MCTLG-5**	**BH-393**	**BH-902**	**BH-946**	**BH-959**
**Cereal grain**															
L*	63.38 ± 0.66^A^	62.85 ± 0.71^A^	61.33 ± 0.71^A^	61.60 ± 0.75^A^	58.10 ± 0.43^ABC^	59.95 ± 0.65^AB^	53.05 ± 4.07^BC^	51.52 ± 4.13^C^	51.69 ± 4.83^C^	53.59 ± 4.05^BC^	51.23 ± 3.89^C^	56.34 ± 0.51^ABC^	56.35 ± 2.34^ABC^	61.53 ± 0.74^A^	57.14 ± 1.08^ABC^
a*	6.86 ± 0.15^A^	7.56 ± 0.13^A^	6.74 ± 0.06^A^	7.27 ± 0.16^A^	7.43 ± 0.12^A^	6.87 ± 0.06^A^	4.73 ± 0.56^B^	4.73 ± 0.59^B^	4.76 ± 0.56^B^	4.83 ± 0.72^B^	4.71 ± 0.51^B^	4.91 ± 0.21^B^	5.10 ± 0.69^B^	5.29 ± 0.70^B^	5.28 ± 0.44^B^
b*	22.54 ± 0.48^A^	23.62 ± 0.39^A^	20.56 ± 0.17^AB^	22.96 ± 0.47^A^	19.68 ± 0.25^ABC^	18.75 ± 0.67^ABCD^	13.80 ± 3.23^D^	14.47 ± 2.69^D^	13.88 ± 2.87^D^	14.76 ± 2.63^CD^	13.70 ± 2.28^D^	16.37 ± 0.50^BCD^	16.22 ± 1.93^BCD^	18.71 ± 0.74^ABCD^	17.05 ± 0.29^BCD^
H^0^	73.06 ± 0.38^AB^	72.26 ± 0.17^ABC^	71.85 ± 0.29^ABC^	72.42 ± 0.17^ABC^	69.31 ± 0.21^C^	69.87 ± 0.64^BC^	70.72 ± 2.45^ABC^	71.76 ± 1.42^ABC^	70.84 ± 1.98^ABC^	71.80 ± 0.87^ABC^	70.85 ± 1.99^ABC^	73.29 ± 0.67^AB^	72.56 ± 0.56^ABC^	74.25 ± 1.64^A^	72.80 ± 1.46^ABC^
C*	23.56 ± 0.47^AB^	24.80 ± 0.41^AB^	21.63 ± 0.14^ABC^	24.08 ± 0.50^AB^	21.03 ± 0.27^ABC^	19.96 ± 0.64^ABCD^	14.59 ± 3.22^E^	15.22 ± 2.73^DE^	14.68 ± 2.88^E^	15.54 ± 2.72^DE^	14.49 ± 2.29^E^	17.09 ± 0.50^CDE^	17.01 ± 2.04^CDE^	19.45 ± 0.85^BCDE^	17.85 ± 0.26^CDE^
**Cereal flour**															
L*	92.40 ± 0.19^ABC^	93.49 ± 0.52^AB^	90.96 ± 0.56^BC^	93.80 ± 0.30^A^	91.02 ± 0.50^BC^	89.96 ± 1.00^C^	73.50 ± 0.38^GH^	72.00 ± 0.27^H^	73.71 ± 0.43^GH^	72.98 ± 0.90^FG^	75.82 ± 0.48^FG^	73.15 ± 0.82^FGH^	76.37 ± 0.39^E^	76.62 ± 0.14^EF^	76.20 ± 1.46^D^
a*	0.38 ± 0.02^E^	0.22 ± 0.02^F^	0.51 ± 0.04^E^	0.19 ± 0.02^F^	0.52 ± 0.02^E^	0.54 ± 0.06^E^	1.33 ± 0.06^A^	1.53 ± 0.04^CD^	1.31 ± 0.01^C^	1.37 ± 0.10^B^	0.79 ± 0.02^C^	1.02 ± 0.02^DE^	1.02 ± 0.19^E^	0.95 ± 0.06^F^	0.13 ± 0.07^F^
b*	9.24 ± 0.19^BC^	7.59 ± 0.13^E^	9.57 ± 0.16^B^	8.39 ± 0.17^CD^	11.75 ± 0.10^A^	8.53 ± 0.26^CD^	7.94 ± 0.08^CD^	8.47 ± 0.16^F^	7.95 ± 0.23^FG^	8.17 ± 0.15^DE^	6.56 ± 0.18^F^	6.86 ± 0.13^G^	6.38 ± 0.30^FG^	6.70 ± 0.24^G^	5.38 ± 0.26^FG^
H^0^	87.62 ± 0.14^ABC^	88.34 ± 0.15^AB^	86.97 ± 0.25^BC^	88.72 ± 0.11^A^	87.48 ± 0.12^ABC^	86.41 ± 0.31^C^	80.49 ± 0.34^FG^	79.74 ± 0.09^G^	80.67 ± 0.18^EFG^	80.51 ± 0.62^FG^	83.13 ± 0.10^D^	81.57 ± 0.05^EF^	80.92 ± 1.34^EFG^	81.96 ± 0.25^DE^	88.64 ± 0.69^A^
C*	9.25 ± 0.19^B^	7.59 ± 0.13^D^	9.58 ± 0.16^B^	8.39 ± 0.17^C^	11.76 ± 0.09^A^	8.55 ± 0.27^C^	8.15 ± 0.08^CD^	8.60 ± 0.16^C^	8.06 ± 0.23^CD^	8.29 ± 0.16^C^	6.60 ± 0.19^E^	6.94 ± 0.14^GE^	6.47 ± 0.32^FE^	6.76 ± 0.24^E^	5.38 ± 0.26^F^
**Cereal gluten**															
L*	49.63 ± 1.55^ABCD^	50.37 ± 1.61^ABCD^	54.36 ± 1.95^A^	48.70 ± 1.50^BCD^	53.29 ± 1.29^AB^	52.30 ± 1.05^ABC^	46.37 ± 1.39^DE^	51.52 ± 4.13^ABCD^	42.17 ± 1.37^EFG^	46.92 ± 1.74^CDE^	42.89 ± 1.20^EF^	38.27 ± 2.34^FGH^	36.35 ± 2.34^HI^	31.53 ± 0.74^I^	37.14 ± 1.08^GH^
a*	0.05 ± 0.03^E^	0.04 ± 0.03^E^	0.21 ± 0.07^CDE^	0.19 ± 0.10^CDE^	0.20 ± 0.09^CDE^	0.18 ± 0.03^DE^	2.44 ± 0.29^A^	2.32 ± 0.09^ABC^	2.21 ± 0.04^ABCD^	2.48 ± 0.08^A^	2.38 ± 0.15^AB^	0.37 ± 0.08^ABCDE^	0.53 ± 0.12^ABCDE^	0.30 ± 0.07^BCDE^	1.99 ± 2.71^ABCDE^
b*	5.88 ± 0.49^F^	6.62 ± 0.27^EF^	7.98 ± 0.80^CDE^	10.06 ± 0.23^B^	11.97 ± 0.22^A^	8.95 ± 0.37B^CD^	6.94 ± 0.19^EF^	10.28 ± 0.73^B^	7.44 ± 0.28^DEF^	8.16 ± 0.34^CDE^	9.56 ± 0.29^BC^	2.77 ± 0.64^G^	2.55 ± 0.46^G^	2.37 ± 1.16^G^	2.75 ± 0.82^G^
H^0^	89.50 ± 0.31^A^	89.65 ± 0.24^A^	88.52 ± 0.38^A^	88.90 ± 0.60^A^	89.06 ± 0.46^A^	88.85 ± 0.23^A^	70.67 ± 1.98^AB^	77.22 ± 1.30^AB^	73.45 ± 0.38^AB^	73.08 ± 0.17^AB^	76.02 ± 1.21^AB^	82.32 ± 0.75^AB^	78.20 ± 2.37^AB^	81.29 ± 5.74^AB^	64.64 ± 2.54^AB^
C*	5.88 ± 0.49^EF^	6.62 ± 0.27^DE^	7.98 ± 0.80^CDE^	10.07 ± 0.23^ABC^	11.97 ± 0.22^A^	8.96 ± 0.37B^CD^	7.36 ± 0.23^DE^	10.54 ± 0.69^AB^	7.76 ± 0.28^CDE^	8.53 ± 0.34^BCD^	9.85 ± 0.25^ABC^	2.80 ± 0.64^G^	2.61 ± 0.46^AB^	2.40 ± 1.14^G^	3.65 ± 2.29^FG^

*Mean ± SD with different superscripts in a row differ significantly (p ≤ 0.05); n = 3 for each treatment*.

H^0^ indicated the color type (angle from 0° to 360°, 0° for red, 90° for yellow, 180° for green, and 270° for blue) of the cereal grains. The range of the H^0^ value fell in the first quadrant of the color wheel (0 refer to red and 90° to yellow color) where wheat (69.31–73.06°), rye (70.72–71.80°), and barley (72.56–74.25°) cultivars were found to overlap in the range 69.31–74.25°. This suggested that the color of grains was predominantly rich in yellow tint with a minor red tint. The relative saturation/purity of grain color was further assigned by C^*^ values which were observed in the range 14.49–24.80. The order of C^*^ values followed the pattern: wheat (19.96–24.80) > barley (17.01–19.45) > rye (14.49–15.54) indicating a relatively greater intensity of yellow tint in wheat followed by barley and rye grains. The values of both H^0^ and C^*^ were found to be statistically different (*p* ≤ 0.05) at inter- and intra-cultivar levels except the H^0^ value in rye cultivars which was not significant (*p* ≥ 0.05) at the intra-cultivar level. The values for different color parameters were found in to be harmony with wheat (16) and rye (25), however, a little variation was observed in the case of barley (28). Variations in grain color of the investigated cereals might be due to differences in the proportion of pigments such as carotenoids, anthocyanins, flavonoids, some tannin, and phenolic compounds along with variation in grain genotype and growing conditions (29).

Cereal flours exhibited a similar trend in their L^*^values (Supplementary Figure 1; Figure 1) following the order: wheat (89.96–93.80) > barley (73.15–76.62) > rye (72.00–75.82). As expected, the L^*^ values of different flours were relatively higher compared to their respective cereal grains. Wheat flour was found to be brighter compared to barley and rye flours. Within cultivars, wheat flour HPW-236 (93.80) was the brightest while rye flour MCTLG-2 (72.00) was relatively darker. The apparent color tint present in different cereal flours as suggested by a^*^ and b^*^ followed the order: rye (0.79–1.53) > barley (0.13–1.02) > wheat (0.19–0.52) and wheat (7.59–11.75) > rye (6.56–8.47) > barley (5.38–6.86), respectively. The higher a^*^ and b^*^ values in barley and rye cultivars compared to wheat cultivars may suggest a higher amount of pigments in these flours or higher ash content. Within cultivars, rye flour from MCTLG-2 was found to possess the highest a^*^ value while wheat flour from HPW-236 possessed the lowest. On the other hand, the highest b^*^ value was observed in wheat cultivar HPW-249, and the lowest in barley cultivar BH-959. The magnitude of a^*^ and b^*^ of the investigated flours was observed to be less compared to their corresponding cereal grains. This could be attributed to the fact that the color of the grain is primarily related to their bran color which gets removed during milling. Statistically, both a^*^ and b^*^ were significantly (*p* ≤ 0.05) different at inter and intra levels of cultivars. The H^0^ values of cereal flours were found to lie in the range of 79.74–88.64°. The different cereal flours had overlapping H^0^ values such as barley (80.92–88.64°), wheat (86.41–88.72°), and rye (79.74–83.13°). These H^0^ values were very close to 90° in the color wheel and correspond to yellow color. In cereal flours, wheat flour HPW-236 was found to possess a greater H^0^ value while rye flour MCTLG-2 possessed the lower H^0^ value. Further, C^*^ in conjunction with H^0^, indicated a decrease in the intensity of yellow tint in the order: wheat (7.59–11.76) > rye (6.60–8.60) > barley (5.38–6.94). Both H^0^ and C^*^ values of the different cereal flours were found to vary significantly (*p* ≤ 0.05) at inter as well as intra-cultivar levels. The variation in the flour color of different cereal grains is mainly attributed to the variation in moisture content, ash content, particle size distribution, flour defilation with bran during milling, and to some extent to phenolic compounds and inherent pigments of grain such as flavonoids, carotenoids, and anthocyanins (30). The obtained results are in agreement with a previous study (16) which reported an L^*^ value of 90.82–92.88; a^*^: 0.22–0.57; b^*^: 7.71–10.8; H^0^: 86.51–88.54°; and C^*^: 7.71–10.80 in the flour of North Indian wheat cultivars. Warechowska et al. (25) reported relatively lower L^*^ values (47.8–51.3) and higher a^*^ (3.29–5.24) and b^*^ (17.99–19.88) values for different rye cultivars. Yeung and Vasanthan (31) reported similar a^*^ (0.4–1.4) and b^*^ (4.2–10.5) values but relatively higher L^*^ values (87.0–93.7) than our results for different barley cultivars.

The gluten derived from different cereal flours using standard protocol was analyzed for its color characteristics (Figure 1) which provides important insights regarding the nature of components and their related oxidative changes (32). The L^*^ values of different cereal glutens were observed to decrease in the order: wheat (48.70–54.36) > rye (42.17–51.52) > barley (31.53–38.27) indicating high relative brightness of wheat glutens as compared to rye and barley glutens (Table 2). Within cultivars, wheat gluten in HPW-155 (54.36) was brighter while barley gluten in BH-946 (31.53) was relatively darker in color. The dark color of barley and rye glutens is possibly due to the relatively higher content of ash and fiber in these cultivars as compared to wheat glutens (33). Secondly, the oxidation of unsaturated compounds such as polyphenols, etc. present in rye and barley gluten could be the other reason for their dark shade.

The a^*^ and b^*^ values of different glutens followed the pattern: rye (2.21–2.48) > barley (0.30–1.99) > wheat (0.04–0.21) and wheat (5.88–11.97) > rye (6.94–10.28) > barley (2.37–2.77), respectively. This trend suggested that the presence of substantial red tint and less yellow tint in rye and barley gluten (Figure 1) as compared to wheat gluten was probably due to high ash and fiber content (33, 34) in these cultivars. At the cultivar level, gluten from rye cultivar MCTLG-4 had the highest a^*^ value while wheat cultivar HPW-147 had the lowest. The highest b^*^ was observed in wheat cultivar HPW-249, and the lowest was seen in barley cultivar BH-946. Both a^*^ and b^*^ values of gluten exhibited significant (*p* ≤ 0.05) differences except for barley where the b^*^ value showed a non-significant difference at the intra-cultivar level. Similar to cereal flours, their derived glutens also exhibited a H^0^ value in the range 64.64–88.52° which belongs to the yellow quadrant of the color wheel. The relatively high value of H^0^ in wheat gluten (88.52–86.65°) indicated a greater degree of yellowness as compared to barley (64.64–82.32°) and rye (70.67–77.22°) gluten. Similarly, C^*^ of cereal glutens followed the order: wheat (5.88–11.97) > rye (7.36–10.54) > barley (2.40–3.65) implying a decrease in intensity of yellow tint from wheat to barley. Furthermore, C^*^ values of cereal glutens were observed to be statistically different (*p* ≤ 0.05) at inter as well as intra-cultivar levels. However, H^0^ of wheat gluten was significantly different (*p* ≤ 0.05) from rye and barley (*p* ≥ 0.05) while at the intra-cultivar level, H^0^ of cereal glutens showed a non-significant (*p* ≥ 0.05) difference.

### Proximate Composition

The proximate composition of the investigated cereal flours is provided in Table 3. The total moisture content of the studied flours was found to be in the range of 6.60–9.75%. Similar results have also been observed in earlier studies where the moisture content of wheat, rye, and barley flour varied in the range of 5.83–15.30% (10, 35). The protein, fat, ash, crude fiber, carbohydrates, and energy of the cereal flours varied from 7.09 to 12.34%, 1.15 to 1.83%, 0.17 to 1.30%, 0.36 to 2.05%, 74.22 to 83.39%, and 357.84 to 378.35 kcal/100 g, respectively. Among cereals, wheat (10.18–11.25%) and rye (7.98–11.37%) flours contained a relatively greater proportion of proteins as compared to barley flours (7.09–9.04%) except BH-902 (12.34%). The ash (A) and crude fiber (CF) content of the cereal flours was observed to decrease in the order: rye (A, 0.70–1.30%; CF, 0.93–2.05%) > barley (A, 0.24–0.50%; CF, 0.50–0.85%) > wheat (A, 0.17–0.40%; CF, 0.36–0.69%) which corresponds to the highest ash and crude fiber content in rye followed by barley and wheat flour.

**Table 3:** Proximate composition and gluten properties of flours of wheat, rye, and barley cultivars.

**Cereal/ cultivar**	**Moisture (%)**	**Protein (%)**	**Fat (%)**	**Fiber (%)**	**Ash (%)**	**Total CHO (%)**	**Energy (kcal/100 g)**	**WG (%)**	**DG (%)**
**Wheat flour**									
HPW-42	7.60 ± 0.24^CDE^	10.37 ± 1.59^AB^	1.46 ± 0.04^ABC^	0.34 ± 1.17^BC^	0.54 ± 0.07^B^	79.30 ± 1.17^ABCDE^	371.83 ± 1.56^ABCD^	20.6 ± 1.30^EF^	7.16 ± 0.66^D^
HPW-147	7.72 ± 0.08^CDE^	11.12 ± 1.78^AB^	1.26 ± 0.23^BC^	0.28 ± 1.83^D^	0.36 ± 0.06^B^	79.25 ± 1.83^ABCDE^	372.84 ± 1.87^ABCD^	29.69 ± 1.13^D^	9.78 ± 0.37^BC^
HPW-155	7.83 ± 0.44^CD^	10.81 ± 1.21^AB^	1.34 ± 0.28^AB^	0.40 ± 0.72^CD^	0.58 ± 0.08^B^	78.94 ± 0.72^ABCDE^	371.02 ± 3.68^ABCD^	29.14 ± 0.86^D^	9.44 ± 0.45^C^
HPW-236	7.84 ± 0.14^CD^	10.18 ± 1.39^AB^	1.39 ± 0.14^AB^	0.17 ± 1.44^D^	0.43 ± 0.03^B^	79.99 ± 1.44^ABCD^	373.17 ± 0.79^ABCD^	41.24 ± 1.81^AB^	11.24 ± 0.73^AB^
HPW-249	8.25 ± 0.40^BCD^	11.25 ± 1.56^AB^	1.15 ± 0.10^C^	0.18 ± 1.99^D^	0.53 ± 0.10^B^	78.64 ± 1.99A^BCDE^	369.94 ± 1.53^BCD^	26.70 ± 0.56^DE^	8.43 ± 0.21^DE^
HPW349	7.92 ± 0.06^CD^	11.09 ± 1.60^AB^	1.42 ± 0.09^ABC^	0.25 ± 1.59^D^	0.69 ± 0.08^B^	78.63 ± 1.59^ABCDE^	371.63 ± 0.91^ABCD^	27.90 ± 0.85^D^	9.74 ± 0.16^BC^
**Rye flour**									
MCTLG-1	8.71 ± 0.39^ABC^	10.51 ± 2.66^AB^	1.77 ± 0.07^AB^	0.91 ± 2.64^AB^	1.01 ± 0.53^B^	77.09 ± 2.64^BCDE^	366.29 ± 0.80^DE^	43.58 ± 2.19^AB^	12.28 ± 0.50^A^
MCTLG-2	8.71 ± 0.19^ABC^	7.98 ± 1.60^AB^	1.73 ± 0.07^AB^	1.24 ± 1.21^A^	2.03 ± 0.40^A^	78.31 ± 1.21^ABCDE^	360.070 ± 2.55^EF^	36.33 ± 2.76^C^	11.24 ± 0.25^AB^
MCTLG-3	9.75 ± 0.34^A^	10.46 ± 0.81^AB^	1.79 ± 0.05^A^	1.20 ± 1.53^A^	0.93 ± 0.50^B^	75.87 ± 1.53^DE^	361.45 ± 2.43^EF^	45.89 ± 3.23^A^	12.75 ± 0.45^A^
MCTLG-4	9.35 ± 0.28^AB^	11.37 ± 1.31^AB^	1.72 ± 0.50^AB^	1.30 ± 1.33^A^	2.05 ± 0.34^A^	74.22 ± 1.33^E^	357.84 ± 4.61^F^	39.27 ± 1.61^BC^	11.46 ± 0.99^A^
MCTLG-5	8.59 ± 0.90^ABC^	11.04 ± 1.80^AB^	1.65 ± 0.02^ABC^	0.70 ± 2.87^BC^	1.04 ± 0.28^B^	76.97 ± 2.87^CDE^	366.93 ± 4.50^CDE^	40.79 ± 2.54^ABC^	9.81 ± 0.17^BC^
**Barley flour**									
BH-393	7.28 ± 0.45^DE^	7.82 ± 1.81^AB^	1.54 ± 0.01^ABC^	0.27 ± 1.60^D^	0.83 ± 0.24^B^	82.26 ± 1.60^AB^	374.19 ± 2.61^ABC^	15.57 ± 3.32^F^	2.07 ± 0.49^FG^
BH-902	7.10 ± 0.61^DE^	12.34 ± 1.12^A^	1.64 ± 0.06^ABC^	0.50 ± 0.43^CD^	0.84 ± 0.25^B^	77.59 ± 0.43^BCDE^	374.44 ± 3.31^ABC^	17.90 ± 2.29^F^	4.17 ± 0.82^E^
BH-946	7.13 ± 0.45^DE^	9.04 ± 1.82^AB^	1.67 ± 0.04^ABC^	0.27 ± 2.06^D^	0.50 ± 0.07^B^	81.39 ± 2.06^ABC^	376.75 ± 1.19^AB^	17.57 ± 3.05^F^	3.29 ± 0.39^EF^
BH-959	6.60 ± 0.16^E^	7.09 ± 1.78^B^	1.83 ± 0.06^A^	0.24 ± 1.92^D^	0.85 ± 0.22^B^	83.39 ± 1.92^A^	378.35 ± 0.18^A^	7.70 ± 2.37^G^	1.51 ± 0.54^G^

*CHO, carbohydrate; WG, wet gluten; DG, dry gluten*.

*Mean ± SD with different superscripts in a column differ significantly (p ≤ 0.05); n = 3*.

Significant (*p* ≤ 0.05) differences were observed in the moisture, fat, fiber, carbohydrate, and energy profile of the cereal flours at inter and intra-cultivar levels. Protein and ash content of flours differ significantly among the three cereals, however, a significant (*p* ≤ 0.05) difference at the intra-cultivar level was observed only in barley and rye cultivars. Our findings on wheat flour are in line with Siddiqi et al. (16), however, Drakos et al. (10) reported a higher fat content in rye and barley flours (5.04–6.14%) as compared to our results (1.54–1.83%) which ultimately led to higher carbohydrate and energy values.

### Flour Performance Properties

The solvent retention capacities (SRC) of different cereal flours are presented in Table 4. Standard solvent systems, i.e., 5% lactic acid (LA), 5% sodium carbonate (SC), and 50% sucrose (Su) solutions were utilized for the approximate prediction of glutenin (solvent-accessible protein), amylopectin (damaged starch), and arabinoxylan (pentosan contents) of cereal flours, respectively (17).

**Table 4:** The solvent retention capacity (SRC) and flour performance properties of wheat, rye, and barley cultivars.

**Cereal**	**SC-SRC (%)**	**LA-SRC (%)**	**Su-SRC (%)**	**W-SRC (%)**	**GPI**	**SDS-SV (ml)**	**WHC (g/g)**	**OHC (g/g)**
**Wheat flour**								
HPW-42	88.99 ± 1.59^FG^	93.63 ± 1.01^E^	107.25 ± 3.44^DE^	82.64 ± 3.65^CDE^	0.48 ± 0.02^CD^	55.00 ± 1.41^D^	1.67 ± 0.01^GH^	1.85 ± 0.01^GHI^
HPW-147	94.64 ± 1.82^DE^	100.29 ± 2.25^CD^	99.40 ± 1.93^EF^	80.84 ± 1.90^EF^	0.52 ± 0.01^AB^	60.00 ± 0.00^C^	1.56 ± 0.01^I^	1.79 ± 0.01^J^
HPW-155	96.46 ± 0.86^DE^	104.60 ± 1.31^BC^	110.74 ± 3.87^CD^	76.15 ± 2.21^F^	0.50 ± 0.01^BC^	68.00 ± 0.00^B^	1.72 ± 0.02^G^	1.90 ± 0.01^FGH^
HPW-236	85.31 ± 1.03^G^	95.86 ± 0.73^DE^	88.21 ± 2.66^G^	79.37 ± 1.09^EF^	0.55 ± 0.01^A^	47.00 ± 1.41^E^	1.56 ± 0.02^I^	1.84 ± 0.02^IJ^
HPW-249	98.84 ± 0.69^CD^	111.92 ± 0.72^A^	120.06 ± 3.29^C^	88.70 ± 1.60^BC^	0.51 ± 0.01^BC^	48.50 ± 0.71^E^	1.64 ± 0.01^H^	1.88 ± 0.01^FGHI^
HPW-349	96.93 ± 1.64^DE^	107.26 ± 3.31^AB^	102.12 ± 3.68^DE^	87.28 ± 1.24^BCD^	0.54 ± 0.01^AB^	72.00 ± 0.00^A^	1.68 ± 0.01^GH^	1.85 ± 0.01^HI^
**Rye flour**								
MCTLG-1	97.69 ± 0.86^CD^	59.23 ± 1.23^HI^	118.56 ± 3.94^C^	91.05 ± 1.75^AB^	0.27 ± 0.01^H^	23.00 ± 0.00^H^	2.03 ± 0.03^BC^	2.09 ± 0.02^D^
MCTLG-2	92.45 ± 1.46^EF^	53.78 ± 2.57^IJ^	108.80 ± 3.63^DE^	96.13 ± 1.13^A^	0.27 ± 0.01^HI^	23.00 ± 0.00^H^	1.86 ± 0.01^E^	1.95 ± 0.02^E^
MCTLG-3	102.20 ± 2.40^C^	60.43 ± 2.31^H^	131.25 ± 2.24^B^	81.03 ± 2.50^EF^	0.26 ± 0.01^HI^	22.00 ± 1.41^HI^	2.10 ± 0.01^A^	2.14 ± 0.01^C^
MCTLG-4	135.47 ± 2.78^A^	67.97 ± 1.86^F^	155.50 ± 2.96^A^	87.25 ± 1.49^BCD^	0.23 ± 0.01^IJ^	24.00 ± 0.00^H^	2.01 ± 0.02^C^	1.92 ± 0.01^EF^
MCTLG-5	122.12 ± 0.38^B^	57.91 ± 3.26^HI^	149.80 ± 3.91^A^	82.13 ± 1.90^DEF^	0.21 ± 0.01^J^	31.00 ± 0.00^G^	2.08 ± 0.01^AB^	2.38 ± 0.01^B^
**Barley flour**								
BH-393	70.51 ± 1.45^H^	50.24 ± 2.41^J^	82.32 ± 3.18^GH^	55.67 ± 1.76^G^	0.33 ± 0.02^G^	20.00 ± 0.00^I^	2.04 ± 0.01^BC^	2.57 ± 0.02^A^
BH-902	87.54 ± 1.46^FG^	67.12 ± 1.84^FG^	90.46 ± 2.30^FG^	61.73 ± 2.19^G^	0.38 ± 0.02^F^	38.00 ± 0.00^F^	1.95 ± 0.01^D^	1.96 ± 0.01^E^
BH-946	73.89 ± 2.63^H^	61.49 ± 1.83^GH^	65.03 ± 2.61^I^	31.07 ± 2.21^I^	0.44 ± 0.01^DE^	30.50 ± 0.71^G^	1.83 ± 0.02^E^	1.90 ± 0.01^FG^
BH-959	46.21 ± 2.31^I^	53.55 ± 1.99^IJ^	77.18 ± 2.65^H^	39.75 ± 2.33^H^	0.43 ± 0.02^E^	24.00 ± 0.00^H^	1.77 ± 0.01^F^	1.73 ± 0.02^K^

*SC-SRC, sodium carbonate-solvent retention capacity; LA-SRC, lactic acid-solvent retention capacity; Su-SRC, sucrose-solvent retention capacity; WSRC, water solvent retention capacity; GPI, gluten performance index; SDS-SV, sodium dodecyl sulfate-sedimentation volume; WHC, water holding capacity; OHC, oil holding capacity*.

*Mean ± SD with different superscripts in a column differ significantly (p ≤ 0.05); n = 3 for each treatment*.

LA-SRC of different cereal flours followed the order: wheat (93.63–111.92%) > rye (53.78–67.97%) ≈ barley (50.24–67.12%), which indicated that wheat flours had higher gluten strength as compared to rye and barley flours. In cereal flours, the wheat cultivar HPW-249 possessed the highest while barley cultivar BH-393 had the lowest LA-SRC value (Table 4). Similar findings were observed by Drakos et al. (10) for rye and barley flours. In the case of SC-SRC, an indicator of damaged starch content (mainly amylopectin), followed the order, rye (92.45–135.47%) > wheat (85.31–98.84%) > barley (46.21–87.54%). This implies that rye cultivar flours contain a relatively higher content of damaged starch as compared to wheat and barley flours which may be due to the typical molecular and structural properties of its starch granule, despite identical milling conditions (10, 36). Among rye flours, MCTLG-4 (135.47%) contained the highest while barley flour BH-959 (46.21%) had the lowest SC-SRC value. The findings of SC-SRC directly govern the viscosity of flour-slurry which is generally dependent upon the cereal type (such as the amount of amylopectin, packing of starch granules at the microscopic level) and milling conditions (such as feed rate, tempering/ conditioning) (10). The Su-SRC of the investigated cereal flours followed the order: rye (108.80–155.50%) > wheat (88.21–120.06%) > barley (65.03–90.46%). This indicated that rye flours had a higher arabinoxylan content which resulted in greater swelling of these flours as compared to barley and rye flours. The higher fiber content in rye flours has been reported previously (1, 10).

These SRC variables are consequently affected by milling procedures which in the case of rye cultivars were observed to have a profound effect as compared to wheat and barley cultivars. Similar behavior has also been reported earlier (10) in rye and barley flour and highlighted the role of extensive milling conditions to enhance the extractability of arabinoxylans. The water-SRC value (W-SRC) provides a cumulative effect of protein and carbohydrate content on the absorption capacity of cereal flours and thus gives an overall functional hydration behavior of all flour components (gluten, starch, and pentosans). The W-SRC of different flours followed the order: rye (81.03–96.13%) > wheat (76.15–88.70%) > barley (31.07–61.73%). The observed trend suggests that the proportions of damaged grain structures of starch and non-starch components (such as protein and other cell wall components) were highest in rye flour followed by wheat and barley flours. Here, it is quite possible that the milling process aided the exposure of the macronutrients present in cereal flours and facilitated their interaction with water molecules (10).

The gluten performance index (GPI), a better predictor of gluten strength and overall baking performance, was observed in the range of 0.21–0.55. The GPI of cereal flours followed a decreasing order in wheat (0.48–0.55), barley (0.33–0.44), and rye (0.21–0.27) indicating that wheat flours have higher overall gluten strength, functionality, and baking performance as compared to barley and rye flours. The higher GPI of wheat flour may be the reason for its suitability for bread making and also its ability to be processed into a variety of foods as compared to barley and rye flour with low GPI. Within cereal flour, the highest GPI was found in wheat cultivar HPW-236, and the lowest in rye cultivar MCTLG-5, which suggested that wheat flours had higher gluten strength and lower damaged grain structure while the reverse was true for rye flours. Statistically significant (*p* ≤ 0.05) differences were observed in the SRC profile of studied cereal flours at inter- and intra-cultivar levels. In the case of rye flours, SRC values (except LA-SRC) were found to be relatively higher as compared to wheat flours. This is attributed to the presence of higher pentosan and damaged starch content which are hydrophilic. Oliete et al. (37) reported higher SRC values of rye flour (W-SRC of 101.65–156.11%, Su-SRC of 143.70–214.22%, SC-SRC of 132.60–181.62%, and LA-SRC of 130.29–152.15%) than soft wheat flours (W-SRC of 57.28–83.20%, Su-SRC of 92.11–123.05%, SC-SRC of 73.90–95.34%, and LA-SRC of 89.42–138.66%). Our SRC results for wheat flours are comparable and relatively lower for rye flour as compared to reported values (37). Such variations could be due to differences in the genetic makeup of the cultivars and also in the milling characteristics, specifically in terms of damaging starch and extractable pentosan content.

The sodium dodecyl sulfate-sedimentation volume (SDS-SV) (Table 4) of the investigated cereal flours indicated higher values in the case of wheat (47–72 ml) as compared to rye (22–31 ml) and barley (20–38 ml) flours. The wheat cultivar HPW-349 exhibited the highest while barley cultivar BH-393 exhibited the lowest SV value. Flours with an SV value <30 ml, between 30 and 60 ml, and more than 60 ml are more suitable for making cookies, chapatti/pasta, and bread, respectively (38). Therefore, wheat flours, particularly HPW-155 and HPW-349, were found to be very suitable for making good quality bread, while the other wheat flours were useful for making chapatti/pasta formulations. On the other hand, rye and barley flours would be more suitable for cookie preparation, except MCTLG-5 and BH-902 which could be utilized for making chapattis/pasta. Significant (*p* ≤ 0.05) differences were observed in the SDS-SV of the studied cereal flours at inter- and intra-cultivar levels. The higher SV values of wheat flours along with higher LASRC and GPI implies greater gluten strength of wheat flours as compared to barley and rye which imparts superior baking characteristics to wheat. Sedimentation values of Indian wheat cultivars have been reported in the range of 30–61 ml (38), 58–76 ml (39), and 47–72 ml (in the present case) which mainly depends upon cultivar type and to some extent on agro-climatic conditions. Despite such variations, all wheat flours were found to be most suitable for chapatti/pasta preparations while few wheat flours (such as HPW-147, HPW-155, and HPW-349) may also be suitable for making breads.

The dry gluten (DG) and wet gluten (WG) content in the studied cereal flours followed: rye (DG: 9.81–12.75%, WG: 36.33–45.89%) > wheat (DG: 7.16–11.24%, WG: 20.6–41.24%) > barley (DG: 1.51–4.17%, WG: 7.70–17.90%) (Table 3). At the cultivar level, the rye cultivar MCTLG-3 possessed the highest while barley cultivar BH-959 had the lowest WG and DG content. It is important to note that rye cultivars contain greater WG and DG content despite weaker gluten strength which is reflected by the lower LASRC, GPI, and SDS-SV values for rye cultivars (Table 4; Supplementary Figure 2). In the process of collecting gluten from the studied cereal flours, the wheat gluten had a characteristic elastic, rubbery, and sticky mass while in the case of rye and barley the obtained mass was relatively inelastic and less sticky which may be due to higher gliadin content and lower glutenin in these cereals along with a significant contribution from ash and fiber content (Supplementary Figure 2) (40). The WG and DG for the studied cereal flours were found to vary significantly (*p* ≤ 0.05) at both inter- and intra-cultivar levels. Similar findings have also been reported by previous studies (35, 41) for wheat cultivars where wet and dry gluten was reported in the range of 17.8–47.23%, 5.9–10.1%, and 14.49–43.70%, 5.12–12.82%, respectively which is consistent with our results.

### Amino Acid Composition of Flour Protein

**Figure 2:**
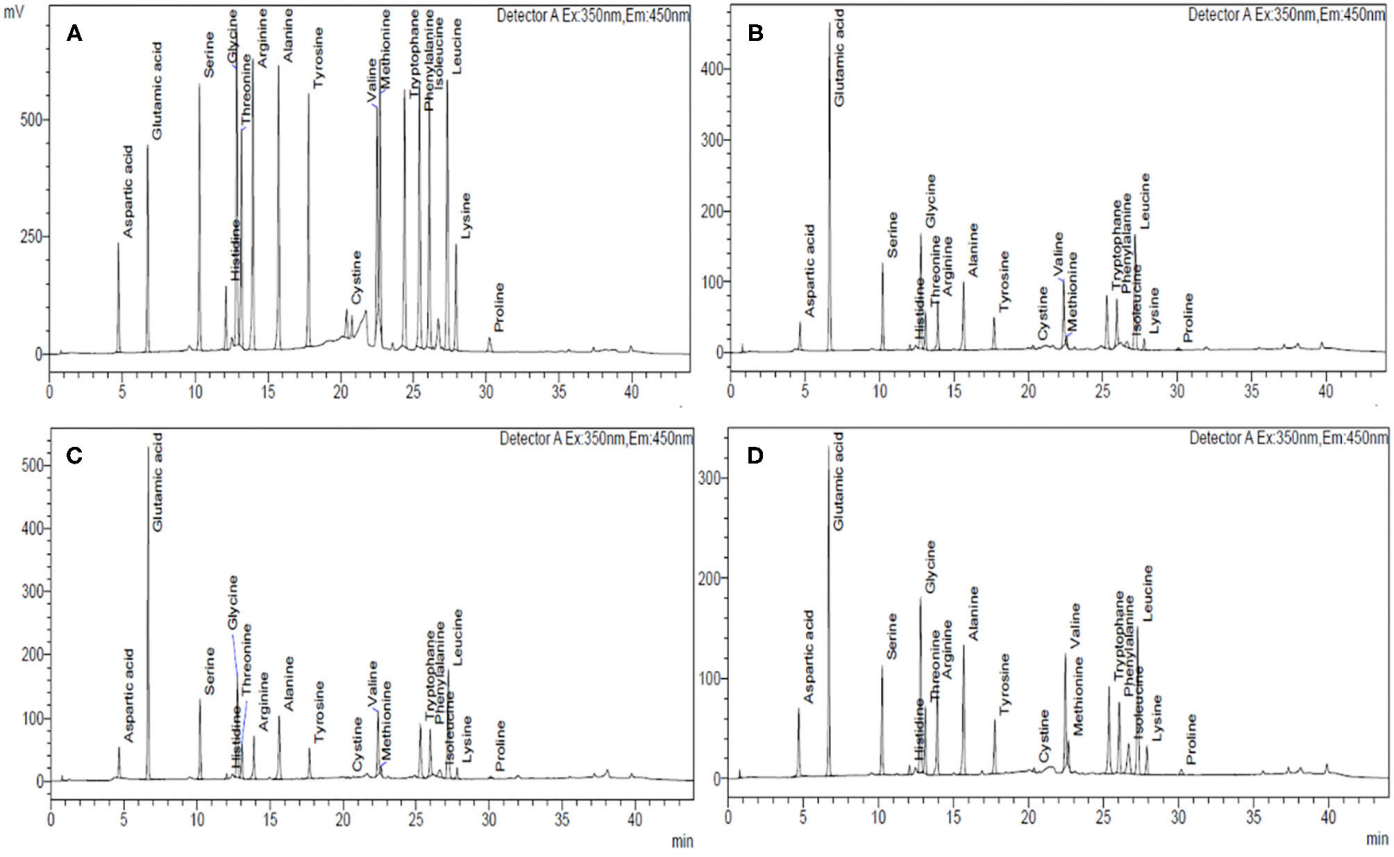
HPLC chromatograms of different cereal flours showing the amino acid composition of **(A)** standard mixture of 18 amino acids, 500 mmol L^−1^; **(B)** wheat, HPW-42, **(C)** rye, MCTLG-1, and **(D)** barley, BH-393.

The amino acid composition and chromatogram of the investigated cereal flours were evaluated using an amino acid analyzer and compared with the established standards (Tables 5A–**C**; Figures 2A–D; Supplementary Figures 3A–L). In different cereals flours, among the essential amino acids (EAA), phenylalanine and leucine were relatively abundant accounting for 4.75–9.22% and 4.40–7.22% of total protein, respectively (Table 5A). Particularly, barley cultivars BH-959 and BH-946 contained the highest amount of phenylalanine (9.22%) and leucine (7.22%) respectively, while wheat cultivars HPW-147 and HPW-155 had relatively lower content of phenylalanine (4.75%) and leucine (4.40%), respectively. Rye (1.97–3.42%) and barley (2.32–2.82%) flours were observed to have the highest amount of limiting EAA, i.e., lysine, as compared to wheat flours (1.09–1.51%) which made barley and rye proteins have higher biological values (BV) as compared to wheat proteins (**Table 5C**). The BV of the cereal flours decreased in the order: barley (85.57–92.75%) > rye (79.79–86.94%) > wheat (65.37–73.03%) which was also supported by the finding of Drakos et al. (10) and Oliete et al. (37). The U.S. Food and Drug Administration (FDA) has also approved barley as a functional food and its commercial utilization for protein fortification. Overall, the total essential amino acids (TEAA) content present in the studied cereal flours followed the order: barley (31.60–32.92%) > rye (29.84–31.45%) > wheat (25.32–27.79%). Further within cereal flours, barley flour (BH-393) possessed the highest while wheat flour HPW-349 possessed the lowest TEAA content. Although, TEAA in rye and barley (29.84–32.92%) was close to the recommended dietary allowances (RDA) prerequisite of the EAA requirement of the Food and Agriculture Organization, FAO for children aged 6 months to 3 years. Similar results have also been reported by Kowieska et al. (9) in wheat, rye, and barley grown in Poland where the distribution of EAA was found to be consistent with our finding. The relative proportion of EAA, threonine, valine, phenylalanine, isoleucine, and leucine was found to exhibit significant (*p* ≤ 0.05) differences while EAA histidine, methionine, and lysine varied non-significantly (*p* ≥ 0.05) at inter- and intra-cultivar levels.

**Table 5A:** Essential amino acid (EAA) composition (g amino acid/100 g protein) in flour of wheat, rye, and barley cultivars.

**Cereal/cultivar**	**His**	**Thr**	**Phe**	**Met**	**Val**	**Ileu**	**Leu**	**Lys**	**TEAA**	**AAS**	**EAAI**	**BV**
**Wheat flour**												
HPW-42	2.66 ± 0.23^A^	3.18 ± 0.03^AB^	5.04 ± 0.48^B^	0.70 ± 0.01^A^	3.79 ± 0.18^B^	3.59 ± 0.50^A^	5.73 ± 0.64^AB^	1.51 ± 0.13^A^	26.19 ± 0.32^C^	79.86 ± 0.98^C^	72.50 ± 0.67^CDE^	67.29 ± 0.73^CDE^
HPW-147	2.75 ± 0.10^A^	3.18 ± 0.03^AB^	4.75 ± 0.88^B^	0.98 ± 0.12^A^	2.93 ± 0.98^B^	4.00 ± 1.08^A^	6.45 ± 1.06^AB^	1.09 ± 0.67^A^	26.13 ± 1.43^C^	79.67 ± 4.35^C^	70.74 ± 0.59^C^	65.37 ± 0.64^E^
HPW-155	2.63 ± 0.13^A^	3.03 ± 0.24^AB^	6.05 ± 0.94^AB^	1.04 ± 0.03^A^	4.26 ± 0.94^AB^	3.48 ± 0.34^A^	4.40 ± 0.84^B^	1.39 ± 0.81^A^	26.27 ± 1.60^C^	80.09 ± 4.87^C^	74.01 ± 6.32B^CDE^	68.95 ± 6.89^BCDE^
HPW-236	2.75 ± 0.38^A^	3.09 ± 0.15^AB^	5.22 ± 0.29^B^	0.95 ± 0.01^A^	3.40 ± 0.21^B^	3.56 ± 0.45^A^	5.56 ± 0.35^AB^	1.37 ± 0.78^A^	25.89 ± 0.25^C^	78.92 ± 0.76^C^	72.74 ± 4.26^CDE^	67.56 ± 4.64^CDE^
HPW-249	2.80 ± 0.34^A^	2.47 ± 0.19^B^	5.64 ± 0.23^B^	1.16 ± 0.14^A^	5.97 ± 0.75^A^	3.43 ± 0.29^A^	5.00 ± 0.11^AB^	1.33 ± 0.15^A^	27.79 ± 1.03^BC^	84.73 ± 3.15^BC^	77.76 ± 0.42^ABCDE^	73.03 ± 0.46^ABCDE^
HPW-349	2.30 ± 0.42^A^	3.06 ± 0.08^AB^	5.17 ± 0.12^B^	0.91 ± 0.29^A^	3.57 ± 0.17^B^	3.75 ± 0.16^A^	5.17 ± 1.20^AB^	1.38 ± 0.17^A^	25.32 ± 1.01^C^	77.18 ± 3.08^C^	71.29 ± 0.73^DE^	65.98 ± 0.80^DE^
**Rye flour**												
MCTLG-1	2.69 ± 0.08^A^	3.53 ± 0.07^AB^	7.42 ± 0.80^AB^	1.29 ± 0.34^A^	3.74 ± 0.51^B^	3.15 ± 0.12^A^	6.06 ± 0.43^AB^	3.10 ± 0.93^A^	30.99 ± 0.58^AB^	94.47 ± 1.78^AB^	90.00 ± 6.05^ABC^	86.37 ± 6.60^ABC^
MCTLG-2	2.53 ± 0.03^A^	3.60 ± 0.46^A^	7.49 ± 0.91^AB^	1.26 ± 0.38^A^	3.67 ± 0.36^B^	3.21 ± 0.19^A^	6.31 ± 0.12^AB^	3.42 ± 1.52^A^	30.50 ± 0.11^AB^	92.98 ± 0.35^AB^	87.18 ± 4.24^ABCDE^	83.30 ± 4.62^ABCDE^
MCTLG-3	2.51 ± 0.06^A^	3.61 ± 0.44^A^	6.81 ± 1.87^AB^	1.22 ± 0.44^A^	3.46 ± 0.26^B^	3.22 ± 0.20^A^	6.49 ± 0.10^AB^	2.51 ± 0.41^A^	29.84 ± 1.08^AB^	90.94 ± 3.28^AB^	85.44 ± 2.67^ABCDE^	81.39 ± 2.91^ABCDE^
MCTLG-4	2.55 ± 0.00^A^	3.64 ± 0.39^A^	7.81 ± 0.46^AB^	1.36 ± 0.24^A^	3.35 ± 0.10^B^	3.23 ± 0.22^A^	6.44 ± 0.03^AB^	3.06 ± 1.05^A^	31.45 ± 0.87^A^	95.88 ± 2.66^A^	90.52 ± 5.37^ABC^	86.94 ± 5.85^ABC^
MCTLG-5	2.59 ± 0.23^A^	3.32 ± 0.18^AB^	8.02 ± 0.04^AB^	1.14 ± 0.01^A^	3.31 ± 0.49^B^	3.37 ± 0.19^A^	6.47 ± 0.17^AB^	1.97 ± 0.06^A^	30.21 ± 0.33^AB^	92.09 ± 1.02^AB^	83.97 ± 0.45^ABCDE^	79.79 ± 0.49^ABCDE^
**Barley flour**												
BH-393	3.17 ± 0.44^A^	3.71 ± 0.44^A^	7.76 ± 1.80^AB^	1.47 ± 0.50^A^	3.45 ± 0.24^B^	3.80 ± 0.26^A^	6.82 ± 0.63^A^	2.76 ± 1.09^A^	32.94 ± 0.05^A^	100.43 ± 0.16^A^	95.21 ± 8.11^A^	92.05 ± 8.84^A^
BH-902	3.09 ± 0.55^A^	3.78 ± 0.34^A^	6.72 ± 0.33^AB^	1.41 ± 0.57^A^	4.15 ± 0.18^AB^	3.83 ± 0.22^A^	6.72 ± 0.48^A^	2.82 ± 0.24^A^	32.53 ± 1.29^A^	99.16 ± 3.94^A^	95.85 ± 9.00^A^	92.75 ± 9.81^A^
BH-946	2.61 ± 0.18^A^	3.25 ± 0.32^AB^	6.71 ± 0.31^AB^	1.43 ± 0.56^A^	4.07 ± 0.30^AB^	3.74 ± 0.33^A^	7.22 ± 0.23^A^	2.35 ± 0.25^A^	31.60 ± 0.53^A^	96.34 ± 1.63^A^	91.39 ± 4.08^AB^	87.89 ± 4.45^AB^
BH-959	2.66 ± 0.11^A^	3.43 ± 0.06^AB^	9.22 ± 0.34^A^	1.00 ± 0.05^A^	3.85 ± 0.00^B^	3.51 ± 0.00^A^	6.91 ± 0.22^A^	2.32 ± 0.29^A^	32.91 ± 0.49^A^	100.33 ± 1.49^A^	89.26 ± 0.57^ABCD^	85.57 ± 0.62^ABCD^
FAO^*^	2	3.1	5.2	2.7	4.3	3.2	6.6	**5.7**	32.8	

*His, histidine; Thr, threonine; Val, valine; Met, methionine; Phe, phenylalanine; Ileu, isoleucine; Leu, leucine; Lys, lysine; TEAA, total essential amino acid; AAS, amino acid score; EAAI, essential amino acid index; BV, biological value*.

*Mean ± SD with different superscripts in a column differ significantly (p ≤ 0.05); n = 3 for each treatment*.

* *represents the data of (19)*.

Glutamine + glutamic acid (Gln + Glu) was observed to be the most abundant non-essential amino acid (NEAA) with the relative mean concentration in the range of 24.92–39.95% followed by asparagine + aspartic acid (Asn + Asp) which was in the range of 3.60–6.60% (Table 5B). Significant (*p* ≤ 0.05) differences were observed in all non-essential amino acids among different cereal cultivars except glycine. Similar results for the mean concentration of NEAA in Polish wheat, barley, and rye cultivars have also been reported by Kowieska et al. (9), except tyrosine which was found to be lower with a mean concentration of 0.97–1.64% in the current study. The total NEAA present in different cereal flours was in the range 67.06–74.68% with wheat cultivar HPW-349 having the highest and barley cultivar BH-393 the lowest proportion of NEAA. It is generally believed that higher concentrations of NEAA like glutamic acid and proline play an important role in dough and baking quality in wheat flour (42–44).

**Table 5B:** Non-essential amino acid (NEAA) composition (g amino acid/100 g protein) in flour of wheat, rye, and barley cultivars.

**Cereal/cultivar**	**Asn + Asp**	**Gln + Glu**	**Ser**	**Gly**	**Arg**	**Ala**	**Tyr**	**Cys**	**Pro**	**NEAA**
**Wheat flour**										
HPW-42	3.77 ± 0.10^AB^	37.17 ± 1.14^A^	5.46 ± 0.58^AB^	3.98 ± 0.16^A^	4.68 ± 0.68^ABC^	3.11 ± 0.31^AB^	3.46 ± 0.34^ABCDE^	1.43 ± 0.27^B^	10.75 ± 2.42^ABCD^	73.80 ± 0.32^A^
HPW-147	4.15 ± 0.24^AB^	39.95 ± 1.90^A^	5.65 ± 0.31^A^	4.14 ± 0.07^A^	4.48 ± 0.39^ABC^	3.06 ± 0.52^AB^	3.45 ± 0.32^ABCDE^	1.53 ± 0.18^AB^	7.47 ± 1.41^D^	73.87 ± 1.43^A^
HPW-155	5.61 ± 0.31^AB^	39.90 ± 0.26^A^	4.79 ± 0.42^AB^	3.07 ± 0.03^A^	4.26 ± 0.09^BC^	3.26 ± 0.62^A^	2.98 ± 0.23^DE^	1.54 ± 0.20^AB^	8.33 ± 1.62^CD^	73.73 ± 1.60^A^
HPW-236	5.53 ± 1.41^AB^	39.32 ± 1.21^A^	5.17 ± 0.29^AB^	3.27 ± 0.26^A^	4.37 ± 0.24^BC^	2.99 ± 0.23^AB^	3.30 ± 0.25^BCDE^	1.29 ± 0.16^B^	8.87 ± 0.99^BCD^	74.11 ± 0.25^A^
HPW-249	3.60 ± 1.16^B^	35.56 ± 1.33^A^	4.59 ± 0.30^AB^	3.36 ± 0.44^A^	3.24 ± 0.43^C^	2.02 ± 0.03^B^	4.18 ± 0.37^A^	1.59 ± 0.06^AB^	14.07 ± 0.02^A^	72.21 ± 1.03^AB^
HPW-349	3.79 ± 0.63^AB^	36.24 ± 1.02^A^	5.08 ± 0.20^AB^	3.95 ± 0.31^A^	5.05 ± 0.87^ABC^	3.18 ± 0.19^A^	3.31 ± 0.13^BCDE^	1.41 ± 0.22^B^	12.68 ± 1.28^ABC^	74.68 ± 1.01^A^
**Rye flour**										
MCTLG-1	5.85 ± 1.17^AB^	25.45 ± 0.76^B^	4.48 ± 0.20^AB^	3.84 ± 0.17^A^	6.24 ± 0.50^AB^	3.91 ± 0.13^A^	3.11 ± 0.12^CDE^	2.30 ± 0.33^AB^	13.83 ± 0.63^AB^	69.01 ± 0.58^BC^
MCTLG-2	5.36 ± 0.29^AB^	27.87 ± 0.47^B^	4.76 ± 0.62^AB^	4.15 ± 0.52^A^	5.90 ± 0.28^AB^	4.07 ± 0.18^A^	2.87 ± 0.16^E^	1.83 ± 0.56^AB^	12.69 ± 0.58^ABC^	69.50 ± 0.12^BC^
MCTLG-3	6.05 ± 1.10^AB^	27.76 ± 0.29^B^	4.73 ± 0.67^AB^	4.16 ± 0.51^A^	5.88 ± 0.54^AB^	3.99 ± 0.29^A^	2.80 ± 0.26^E^	1.67 ± 0.33^AB^	13.14 ± 1.61^ABC^	70.177 ± 1.07^BC^
MCTLG-4	6.60 ± 0.57^A^	27.80 ± 1.06^B^	4.68 ± 0.74^AB^	4.11 ± 0.58^A^	5.81 ± 0.43^AB^	4.01 ± 0.27^A^	2.87 ± 0.16^E^	1.53 ± 0.14^AB^	11.14 ± 2.54^ABCD^	68.55 ± 0.88^C^
MCTLG-5	6.28 ± 0.19^AB^	26.52 ± 2.78^B^	4.30 ± 0.04^AB^	3.79 ± 0.25^A^	6.50 ± 0.63^A^	3.78 ± 0.29^A^	3.10 ± 0.17^CDE^	3.16 ± 0.97^A^	12.36 ± 0.57^ABCD^	69.79 ± 0.33^BC^
**Barley flour**										
BH-393	4.63 ± 0.01^AB^	25.84 ± 0.82^B^	4.42 ± 0.28^AB^	4.02 ± 0.31^A^	5.76 ± 0.54^AB^	3.63 ± 0.17^A^	4.10 ± 0.16^AB^	2.68 ± 0.69^AB^	11.99 ± 0.24^ABCD^	67.06 ± 0.05^C^
BH-902	6.03 ± 0.58^AB^	27.33 ± 1.10^B^	4.04 ± 0.03^AB^	3.87 ± 0.52^A^	5.51 ± 0.18^AB^	3.59 ± 0.23^A^	3.95 ± 0.05^ABC^	1.04 ± 0.21^B^	12.10 ± 0.40^ABCD^	67.47 ± 1.29^C^
BH-946	5.69 ± 0.10^AB^	24.92 ± 0.06^B^	4.30 ± 0.45^AB^	3.91 ± 0.47^A^	5.58 ± 0.29^AB^	3.67 ± 0.12^A^	3.91 ± 0.13^ABC^	2.16 ± 0.04^AB^	13.85 ± 0.81^AB^	68.40 ± 0.54^C^
BH-959	5.96 ± 0.28^AB^	25.88 ± 1.42^B^	3.93 ± 0.06^B^	3.47 ± 0.16^A^	5.59 ± 0.28^AB^	3.43 ± 0.22^A^	3.77 ± 0.04^ABCD^	2.51 ± 0.54^AB^	12.55 ± 0.99^ABCD^	67.09 ± 0.49^C^

*Asn + Asp, asparagine + aspartic acid; Gln + Glu, glutamine + glutamic acid; Ser, serine; Gly, glycine; Arg, arginine; Ala, alanine; Tyr, tyrosine; Cys, cysteine; Pro, proline; TNEAA, total non-essential amino acid*.

*Mean ± SD with different superscripts in a column differ significantly (p ≤ 0.05); n = 3 for each treatment*.

The amino acid concentration of wheat flour is in close agreement with Alijošius et al. (45) and Gálová et al. (46). A slightly higher concentration of proline (11.73–16.93%) has been reported by Šterna et al. (47) in five spring barley cultivars compared to our results. Kihlberg et al. (48) reported a concentration of proline (8.27–9.68%) in rye flour which was slightly lower than our findings. The variation in amino acid composition is largely dependent on the genotype, milling conditions, flour extraction rate, wheat type (soft, hard, semi-soft), growing environmental conditions like CO_2_ concentration, growing temperature application of fertilizers, and protein content of flour, etc. (49–54).

The amino acid score (AAS) indicates the quality of protein in terms of its EAA content to that of the reference protein (19). The AAS score of the cereal flours followed the order: barley (96.34–100.43%) > rye (90.94–95.88%) > wheat (77.18–84.73%). The barley cultivar BH-393 had the highest while the wheat cultivar HPW-349 had the lowest AAS score. Alijošius et al. (45) reported an AAS for wheat (88.63–98.81%) which was slightly higher than the present results and for rye (80.12–89.82%), their results were lower than ours. These variations in AAS values could be due to cultivar differences.

Furthermore, the amino acids (AAs) were classified into three hydropath groups, i.e., hydrophilic, hydrophobic, and neutral amino acids, according to IMGT amino acid classification and are presented in Table 5C. The hydrophobic AAs constituted 22.95–30.43% of the total amino acids and were comprised of aliphatic, S-containing, and some aromatic amino acids which accounted for 15.39–19.07%, 2.12–4.14%, and 4.75–9.22%, respectively. The S-containing amino acids were found to be higher in the rye (2.89–4.30%) and barley (2.46–4.14%) while lower in wheat (2.12–2.74%). Overall total hydrophobic AAs were found to be higher in barley cultivars (27.47–30.43%), followed by rye (26.87–29.26%) and wheat (22.95–24.79%). A significant (*p* ≤ 0.05) difference was observed among the hydrophobic group of amino acids at inter- and intra-cultivar levels, however, in rye cultivars, the aliphatic and aromatic hydrophobic AAs showed a non-significant difference (*p* ≥ 0.05).

**Table 5C:** IMGT (ImMunoGeneTics) amino acid classification in flour of wheat, rye, and barley cultivars (g amino acid/100 g protein).

**Cereal/cultivar**	**Hydrophobic amino acid**				**Hydrophilic amino acid**			**Neutral amino acid**			
	**Aliphatic**	**S-containing**	**Aromatic AA**	**Total hydrophobic**	**Basic AA**	**Acidic**	**Total hydrophilic**	**Non-polar**	**Polar (hydroxyl AA)**	**Aromatic AA**	**Total neutral**
**Wheat flour**											
HPW-42	16.22 ± 1.26^BC^	2.12 ± 0.25^D^	5.04 ± 0.48^B^	23.39 ± 1.03^CDE^	8.85 ± 0.58^BCDE^	40.94 ± 1.24^ABC^	49.79 ± 1.82^AB^	14.73 ± 2.58^ABCD^	8.63 ± 0.62^A^	3.46 ± 0.34^ABCDE^	26.82 ± 2.85^AB^
HPW-147	16.43 ± 0.45^BC^	2.50 ± 0.06^CD^	4.75 ± 0.88^B^	23.69 ± 1.27^BCDE^	8.32 ± 0.96^CDE^	44.10 ± 1.66^ABC^	52.41 ± 2.62^A^	11.62 ± 1.34^CD^	8.83 ± 0.33^A^	3.45 ± 0.32^ABCDE^	23.90 ± 1.35^AB^
HPW-155	15.39 ± 0.37^C^	2.58 ± 0.17^CD^	6.05 ± 0.94^AB^	24.02 ± 0.75^BCDE^	8.28 ± 0.77^DE^	45.51 ± 0.56^A^	53.79 ± 1.33^A^	11.39 ± 1.65^D^	7.81 ± 0.66^A^	2.98 ± 0.23^DE^	22.19 ± 2.08^B^
HPW-236	15.50 ± 0.55^C^	2.24 ± 0.17^D^	5.22 ± 0.29^B^	22.95 ± 0.08^E^	8.49 ± 0.64^CDE^	44.85 ± 0.20^AB^	53.34 ± 0.84^A^	12.15 ± 0.73^BCD^	8.27 ± 0.45^A^	3.30 ± 0.25^BCDE^	23.71 ± 0.92^AB^
HPW-249	16.41 ± 0.97^BC^	2.74 ± 0.08^BCD^	5.64 ± 0.23^B^	24.79 ± 1.13^BCDE^	7.37 ± 0.24^E^	39.16 ± 0.16^CD^	46.53 ± 0.41^BCD^	17.44 ± 0.46^A^	7.06 ± 0.11^A^	4.18 ± 0.37^A^	28.68 ± 0.72^A^
HPW-349	15.67 ± 1.05^C^	2.32 ± 0.07^CD^	5.17 ± 0.12^B^	23.16 ± 1.10^DE^	8.73 ± 1.11^BCDE^	40.03 ± 1.64^BC^	48.76 ± 0.53^ABC^	16.63 ± 0.97^ABC^	8.14 ± 0.28^A^	3.31 ± 0.13^BCDE^	28.08 ± 0.57^AB^
**Rye flour**											
MCTLG-1	16.87 ± 0.16^ABC^	3.60 ± 0.01^ABCD^	7.42 ± 0.80^AB^	27.88 ± 0.96^AB^	12.04 ± 0.51^A^	31.30 ± 0.41^E^	43.33 ± 0.11^D^	17.67 ± 0.47^A^	8.01 ± 0.27^A^	3.11 ± 0.12^CDE^	28.79 ± 0.85^A^
MCTLG-2	17.27 ± 0.23^ABC^	3.09 ± 0.18^ABCD^	7.49 ± 0.91^AB^	27.85 ± 0.86^AB^	10.85 ± 0.15^ABCD^	33.23 ± 0.17^E^	44.09 ± 0.32^CD^	16.84 ± 0.06^AB^	8.35 ± 1.08^A^	2.87 ± 0.16^E^	28.06 ± 1.18^AB^
MCTLG-3	17.18 ± 0.27^ABC^	2.89 ± 0.11^ABCD^	6.82 ± 1.87^AB^	26.87 ± 2.03^ABCDE^	10.91 ± 0.07^ABCD^	33.82 ± 1.39^E^	44.70 ± 1.46^BCD^	17.31 ± 2.12^A^	8.34 ± 1.11^A^	2.81 ± 0.26^E^	28.44 ± 3.49^A^
MCTLG-4	17.04 ± 0.08^ABC^	2.89 ± 0.10^ABC^D	7.81 ± 0.46^AB^	27.74 ± 0.45^ABC^	11.41 ± 0.62^AB^	34.40 ± 0.50^DE^	45.81 ± 1.12^BCD^	15.25 ± 1.96^ABCD^	8.32 ± 1.13^A^	2.87 ± 0.16^E^	26.45 ± 0.67^AB^
MCTLG-5	16.93 ± 0.56^ABC^	4.30 ± 0.98^A^	8.02 ± 0.04^AB^	29.26 ± 0.45^A^	11.06 ± 0.93^ABC^	32.80 ± 2.59^E^	43.86 ± 1.66^CD^	16.15 ± 0.82^ABCD^	7.63 ± 0.22^A^	3.10 ± 0.17^CDE^	26.88 ± 1.21^AB^
**Barley flour**											
BH-393	17.70 ± 0.43^AB^C	4.14 ± 0.19^AB^	7.76 ± 1.80^AB^	29.60 ± 2.43^A^	11.69 ± 0.99^A^	30.47 ± 0.81^E^	42.16 ± 1.80^D^	16.01 ± 0.07^ABCD^	8.13 ± 0.72^A^	4.10 ± 0.16^AB^	28.24 ± 0.63^AB^
BH-902	18.29 ± 0.14^AB^	2.46 ± 0.78^CD^	6.72 ± 0.33^AB^	27.47 ± 0.60^ABCD^	11.42 ± 0.61^AB^	33.37 ± 1.68^E^	44.79 ± 1.07B^CD^	15.98 ± 0.12^ABCD^	7.82 ± 0.31^A^	3.95 ± 0.05^ABC^	27.75 ± 0.47^AB^
BH-946	19.07 ± 0.46^A^	3.76 ± 0.35^ABC^	6.71 ± 0.31^AB^	29.54 ± 0.50^A^	10.96 ± 0.81^ABCD^	30.27 ± 0.43^E^	41.24 ± 0.38^D^	17.84 ± 0.46^A^	7.47 ± 0.24^A^	3.91 ± 0.10^ABC^	29.23 ± 0.12^A^
BH-959	17.70 ± 0.44^ABC^	3.51 ± 0.49^ABCD^	9.22 ± 0.34^A^	30.43 ± 0.29^A^	10.57 ± 0.10^ABCD^	31.84 ± 1.71^E^	42.42 ± 1.61^D^	16.02 ± 1.15^ABCD^	7.36 ± 0.12^A^	3.77 ± 0.04^ABCD^	27.15 ± 1.32^AB^

*Total hydrophobic amino acid: Alanine + cysteine + valine + methionine + tryptophan + phenylalanine + isoleucine + leucine; total hydrophilic amino acid: Asparagine+aspartic acid + glutamine+glutamic acid + arginine + lysine; neutral amino acid: Serine + histidine + glycine + threonine + tyrosine + proline. Total AAS, total amino acid score-; EAAI, essential amino acid index; BV, biological value*.

*Mean ± SD with different superscripts in a column differ significantly (p ≤ 0.05); n = 3 for each treatment*.

The hydrophilic AAs constituted 41.24–53.79% of the total amino acids and were comprised of acidic and basic amino acids which accounted for 30.27–44.85% and 7.37–12.64% of the total amino acids, respectively. A significant (*p* ≤ 0.05) difference was observed among the hydrophilic class of amino acids at inter- and intra-cultivar levels while acidic AAs in barley cultivars showed a non-significant (*p* ≥ 0.05) difference.

The total neutral amino acids of cereal flour varied from 22.19 to 29.23% and constituted non-polar, polar, and aromatic amino acids which accounted for 11.39–17.84%, 7.06–8.83%, and 2.81–4.18% of the total amino acids, respectively. Barley cultivar BH-946 and wheat cultivar HPW-155 had the highest and the lowest content of total neutral AAs, respectively. Statistically, a significant (*p* ≤ 0.05) difference was observed among the neutral AAs, while a non-significant (*p* ≥ 0.05) difference among polar hydroxyl AAs was observed both at inter as well as intra-cultivar levels.

### SDS-PAGE of Wheat, Rye, and Barley Flour

**Figure 3:**
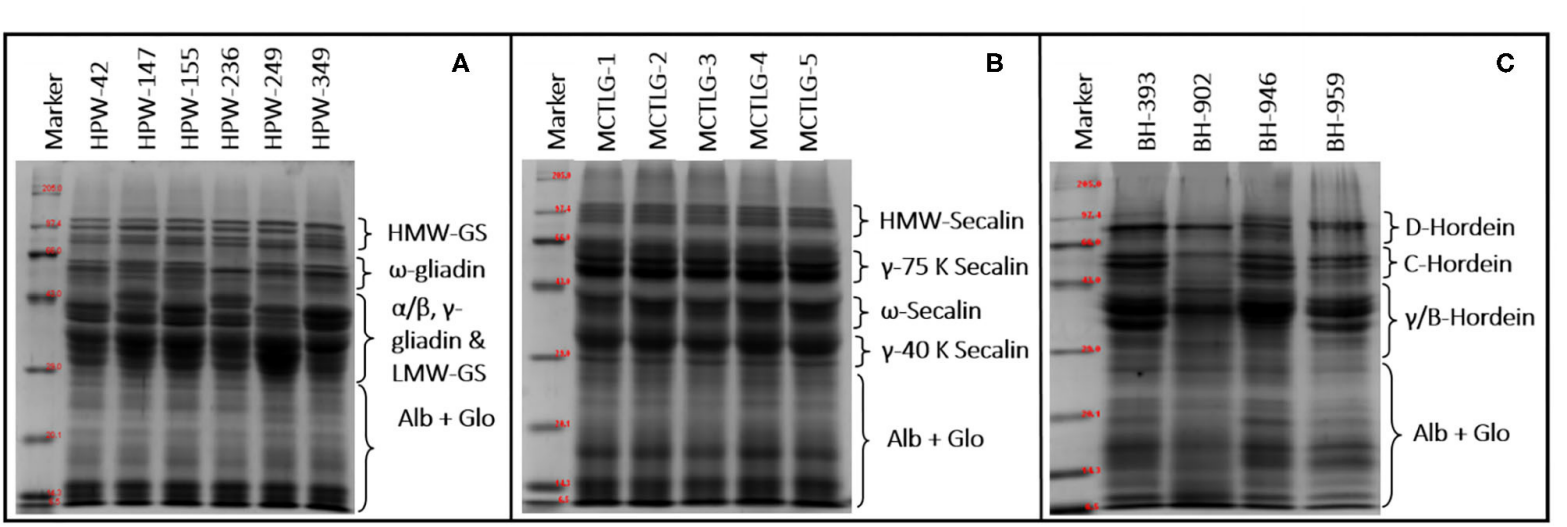
SDS-PAGE of flour proteins from **(A)** wheat, **(B)** rye, and **(C)** barley cultivars under reducing conditions.

The SDS-PAGE pattern of flour proteins present in different cereal cultivars under reduced conditions is given in Figures 3A–C. Many authors have broadly classified the storage proteins of the Triticeae family into three main groups, namely high molecular weight (HMW), medium molecular weight (MMW)/sulfur poor, and low molecular weight (LMW)/sulfur-rich proteins (21, 42, 55).

The HMW group in wheat, rye, and barley comprises HMW-glutenin subunits, HMW-secalin, and D-hordein, respectively. The MMW group comprises of ω-gliadin (wheat), ω-secalin (rye), and C-hordein (barley), while the LMW group contains monomeric proteins (α/β-gliadin, γ-gliadin in wheat, γ-40k-secalin in rye, and γ-hordein in barley) and polymeric proteins (LMW-GS in wheat, γ-75k-secalin in rye, and B-hordein in barley).

The total number of bands in SDS-PAGE ranged from 21 to 24 in wheat, 18 in rye, and 17 to 22 in barley flours. Among high molecular weight (HMW) proteins, the MW of protein-subunits in wheat, rye, and barley ranged between 68.38 and 119.66 kDa (4–5 subunits), 82.33 and 117.78 kDa (4 subunits), and 73.08 and 108.57 kDa (2–4 subunits), respectively. Based on densitometric analysis (Table 6), the HMW group comprised 9.84–18.75% of the total flour proteins with a proportion in the range of 13.57–18.75 kDa in wheat cultivars, 9.84–14.98 kDa in rye cultivars, and 12.78–16.33 kDa in barley cultivars. The wheat cultivar HPW-349 was found to possess the highest HMW proportion, and the rye cultivar MCTLG-3 the lowest. The HMW group mainly comprises glutenin protein which is generally polymeric and, due to the presence of cysteine residues, forms inter- and intra-molecular disulfide linkages. These proteins play an important role in strengthening the three-dimensional structure of the gluten framework by imparting high elastic strength to the dough (56). Previous studies have explained clearly the role of the HMW group in the baking performance of dough by providing an elastic property to the dough (57).

**Table 6:** The relative proportion (%) of total flour proteins in wheat, rye, and barley cultivars.

**Cereal flour**	**HMW (HMW-GS/HMW-secalin/D-hordein)**	**MMW/sulfur-poor (ω-Gliadin/ω-secalin/C-hordein)**	**LMW/sulfur-rich (α/β-, γ-Gliadin/γ-75 k, γ-40 k-secalin/B-hordein)**	**ALB + GLO**
**Wheat flour**				
HPW-42	13.57 ± 0.96^AB^	11.28 ± 0.99^AB^	38.61 ± 1.71^ABCDE^	36.53 ± 0.24^A^
HPW-147	18.03 ± 4.44^AB^	7.00 ± 2.10^B^	39.66 ± 2.43^ABCDE^	35.32 ± 0.09^A^
HPW-155	14.15 ± 1.75^AB^	11.09 ± 0.46^AB^	40.49 ± 0.18^ABCDE^	34.26 ± 2.40^A^
HPW-236	15.60 ± 1.67^AB^	5.73 ± 1.06^B^	35.92 ± 3.26^CDE^	42.75 ± 5.99^A^
HPW-249	17.63 ± 1.37^AB^	8.91 ± 0.43^AB^	37.79 ± 5.09^BCDE^	35.67 ± 3.30^A^
HPW-349	18.75 ± 1.83A	15.08 ± 2.49^A^	41.16 ± 6.32^ABCDE^	25.01 ± 5.66^A^
**Rye flour**				
MCTLG-1	13.13 ± 2.07^AB^	12.92 ± 2.57^AB^	50.14 ± 3.19^ABC^ (14.98 ± 0.63^*^+ 13.86 ± 1.40^**^)	23.81 ± 7.84^A^
MCTLG-2	14.98 ± 0.63^AB^	15.09 ± 2.29^A^	47.06 ± 5.46^ABCD^ (21.20 ± 2.51^*^+ 19.14 ± 4.67^**^)	22.87 ± 8.38^A^
MCTLG-3	9.84 ± 0.60^B^	15.22 ± 3.68^A^	52.46 ± 2.23^AB^ (15.09 ± 2.29^*^+ 14.76 ± 1.58^**^)	22.48 ± 6.51^A^
MCTLG-4	13.86 ± 1.40^AB^	14.76 ± 1.58^A^	46.24 ± 5.08^ABCD^ (25.86 ± 2.95^*^+ 27.10 ± 0.41^**^)	25.14 ± 8.06^A^
MCTLG-5	12.73 ± 4.56^AB^	12.48 ± 2.00^AB^	53.04 ± 2.04^A^ (22.87 ± 8.38^*^+ 25.14 ± 8.06^**^)	21.89 ± 8.60^A^
**Barley flour**				
BH-393	15.69 ± 2.46^AB^	16.60 ± 2.53^A^	29.66 ± 1.30^E^	38.05 ± 6.29^A^
BH-902	12.78 ± 1.40^AB^	14.81 ± 1.42^A^	33.91 ± 1.51^DE^	38.50 ± 1.30^A^
BH-946	16.33 ± 2.12^AB^	16.34 ± 0.85^A^	31.07 ± 1.44^E^	36.26 ± 4.40^A^
BH-959	15.47 ± 0.87^AB^	11.16 ± 1.45^AB^	35.04 ± 6.68^DE^	38.33 ± 4.36^A^

*HMW, high molecular weight; MMW, medium molecular weight; LMW, low molecular weight, ALO + GLO, albumin + globulin*.

*Mean ± SD with different superscripts in a column differ significantly (p ≤ 0.05); n = 2 for each treatment*.

* *refers to 75k-secalin fraction*;

** *refers to 40k-secalin*.

The monomeric protein ω-prolamins, also called sulfur-poor, in wheat cultivars falls in the range between 47.60 and 61.85 kDa corresponding to four protein subunits, except for wheat cultivar HPW-236 where two peptide bands in this region were observed. On the other hand, in the case of barley and rye, the distribution of ω-region ranged from 43.93 to 60.42 kDa (2-4 protein subunits) and 48.93 to 51.67 kDa (1 protein subunit), respectively. The relative proportion of the sulfur-poor group of the studied flours varied between 5.73 and 16.60%, with a proportion of 5.73–15.08% in wheat cultivars, 12.48–15.22% in rye cultivars, and 11.16–16.60% in barley cultivars These ω-proteins usually lack cysteine residue in their peptide chain and are not involved in disulfide bonding (56).

The α/β-gliadins, γ-gliadins, and LMW-GS, also known as sulfur-rich regions, in wheat flours were distributed in the range 27.03–45.24 kDa while barley flours were found to contain γ/B-hordein distributed between 29.00 and 43.63 kDa of the LMW group. The rye flours were observed to be enriched with γ-75k-secalins and γ-40k-secalins having an MW ranging from 53.45 to 66.00 kDa and 28.46 to 40.84 kDa, respectively.

In the studied cereal flours, a high degree of polymorphism was observed in wheat as compared to barley while relatively obscure patterns were noticed in the case of rye flours. The majority of the wheat cultivars (HPW-42, HPW-147, HPW-236, HPW-249) contained 7-8 polypeptides in the LMW region of 27.03–45.24 kDa, however, wheat cultivars HPW-155 and HPW-349 contained 4-6 polypeptide in this region. Similarly, for barley cultivars, 4-6 polypeptides were observed in the range of 29.00–43.63 kDa, barley cultivar BH-393 exhibited 4 polypeptides, and BH-902 exhibited 6 polypeptides while BH-946 and BH-959 exhibited 5 polypeptides each in the LMW region. However, in the case of rye cultivars, the observed patterns were difficult to distinguish from the cultivar type unlike in wheat and barley. The LMW region thus can be used to distinguish wheat and barley cultivars and can act as a biochemical marker. Nonetheless, the regions 27.03–34.6 kDa (α/β gliadin, wheat), 28.46–32.63 (γ-40k-secalins, rye), and 29.00–43.62 (γ/B-hordein, barley) of the LMW group were not good enough to resolve the intense bands into clear individual segregated bands. In the studied cereal flours, the relative proportion of the LMW group was observed to be in the range 29.66–53.04% of total protein. The highest proportion of the LMW group was observed in rye cultivar MCTLG-5 (53.04%) while lowest was seen in barley cultivar BH-393 (29.66%). In general, the LMW group is rich in sulfur proteins mainly involved in inter and intra-molecular disulphide linkages (56). Many studies have demonstrated a positive correlation between the LMW group and bread loaf volume and dough rheological properties (such as development time and stability) (57). The metabolic active proteins (albumin and globulin, ALB + GLO) of wheat, rye, and barley cultivars were observed in the range of 26.93–8.37 kDa, 6.50–26.63 kDa, and 11.15–28.05 kDa, respectively. However, among different cereal cultivars, no polymorphism was observed in albumin and globulin fractions. The relative proportion of ALB + GLO did not show any significant difference (*p* ≥ 0.05). Statistically, a significant (*p* ≤ 0.05) difference was observed among HMW, MMW, and LMW proteins for analyzed cereal grains at inter- and intra-cultivar levels except for barley in the percentage of HMW which only differed at the inter-cultivar level. Our results on the relative proportion of molecular weight distribution (HMW, ω-Prolamin, LMW, and ALB + GLO) were in close agreement with earlier reports (16, 21, 42, 46).

## Conclusion

Comparative evaluation of technological and functional properties of wheat, rye, and barley was carried out. Barley and rye were found to contain proteins of high biological value while wheat flours possessed a better technological property due to higher gluten strength. SDS-SV indicated that wheat cultivars HPW-155 and HPW-349 would be more suitable for making quality bread while its other cultivar was good for chapatti making. Most cultivars of barley and rye were found to be suitable for cookie preparation except MCTLG-5 and BH-902 which can be used for chapatti making. High polymorphism was observed in wheat cultivars as compared to rye and barley. Furthermore, the comparative amino acid analysis of these cereals supported the utilization of barley and rye cultivars for protein fortification owing to their richness in limiting essential amino acids, which is of paramount importance in the manufacturing of composite flours. These insights could be quite resourceful for manufacturers and researchers looking for versatile flour material for the development of healthy food products.

## Data Availability Statement

The raw data supporting the conclusions of this article will be made available by the authors, without undue reservation.

## Author Contributions

MR: methodology, formal analysis, data curation, investigation, writing—original draft preparation, reviewing, and editing. GS: reviewing and editing. RS: formal analysis, reviewing, and editing. DS: conceptualization, supervision, resources, reviewing, editing, and funding acquisition. BG: supervision. MB: statistical analysis. All authors contributed to the article and approved the submitted version.

## Conflict of Interest

The authors declare that the research was conducted in the absence of any commercial or financial relationships that could be construed as a potential conflict of interest.

## Publisher's Note

All claims expressed in this article are solely those of the authors and do not necessarily represent those of their affiliated organizations, or those of the publisher, the editors and the reviewers. Any product that may be evaluated in this article, or claim that may be made by its manufacturer, is not guaranteed or endorsed by the publisher.
