# Supplementary material for: Comparative Quality Evaluation of Physicochemical, Technological, and Protein Profiling of Wheat, Rye, and Barley Cereals
Source: Front Nutr. 2021 Sep 16;8:694679. doi: 10.3389/fnut.2021.694679 (PMC8481659; doi:10.3389/fnut.2021.694679)
Supplement: Supplementary file 1 [file Data_Sheet_1.docx]

**Comparative Quality Evaluation of Physicochemical, Technological and Protein Profiling of Wheat, Rye and Barley Cereals.**

**Supporting Information**

**Annexure 1**


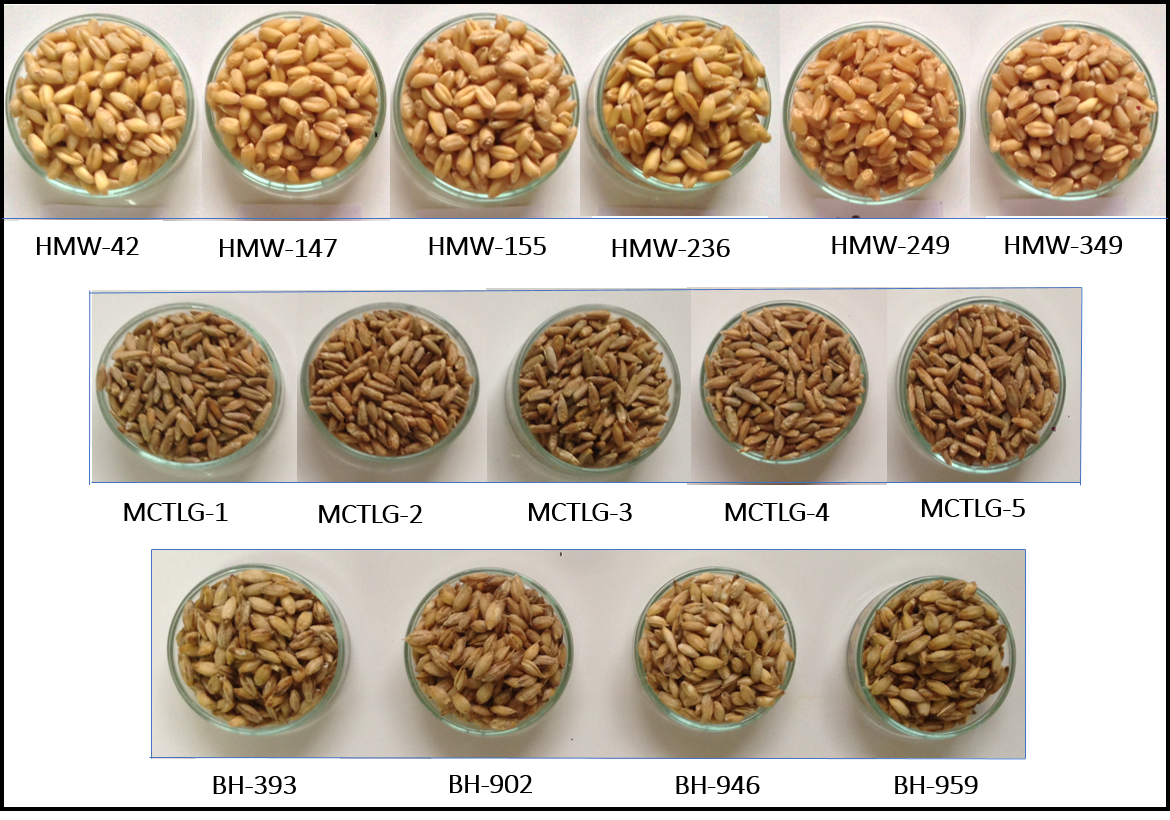


**Fig S1.** Different wheat, rye and barley cultivars used in this study.


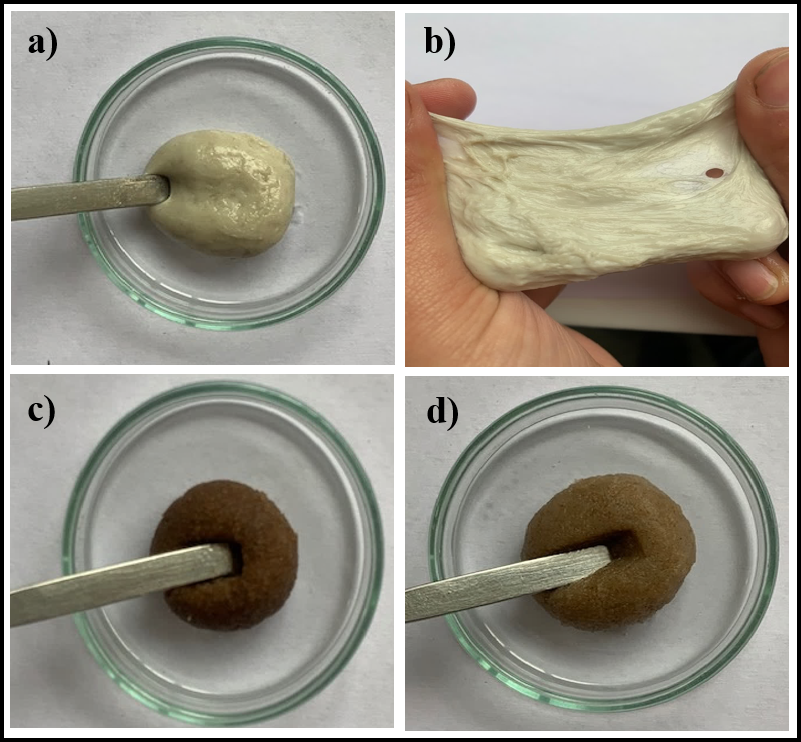


**Fig. S2.** Gluten obtained from different cereals showing their relative elastic behaviour: (a-b) Wheat, HPW-42 c) Rye, MCTLG-1 and d) Barley, BH-393.


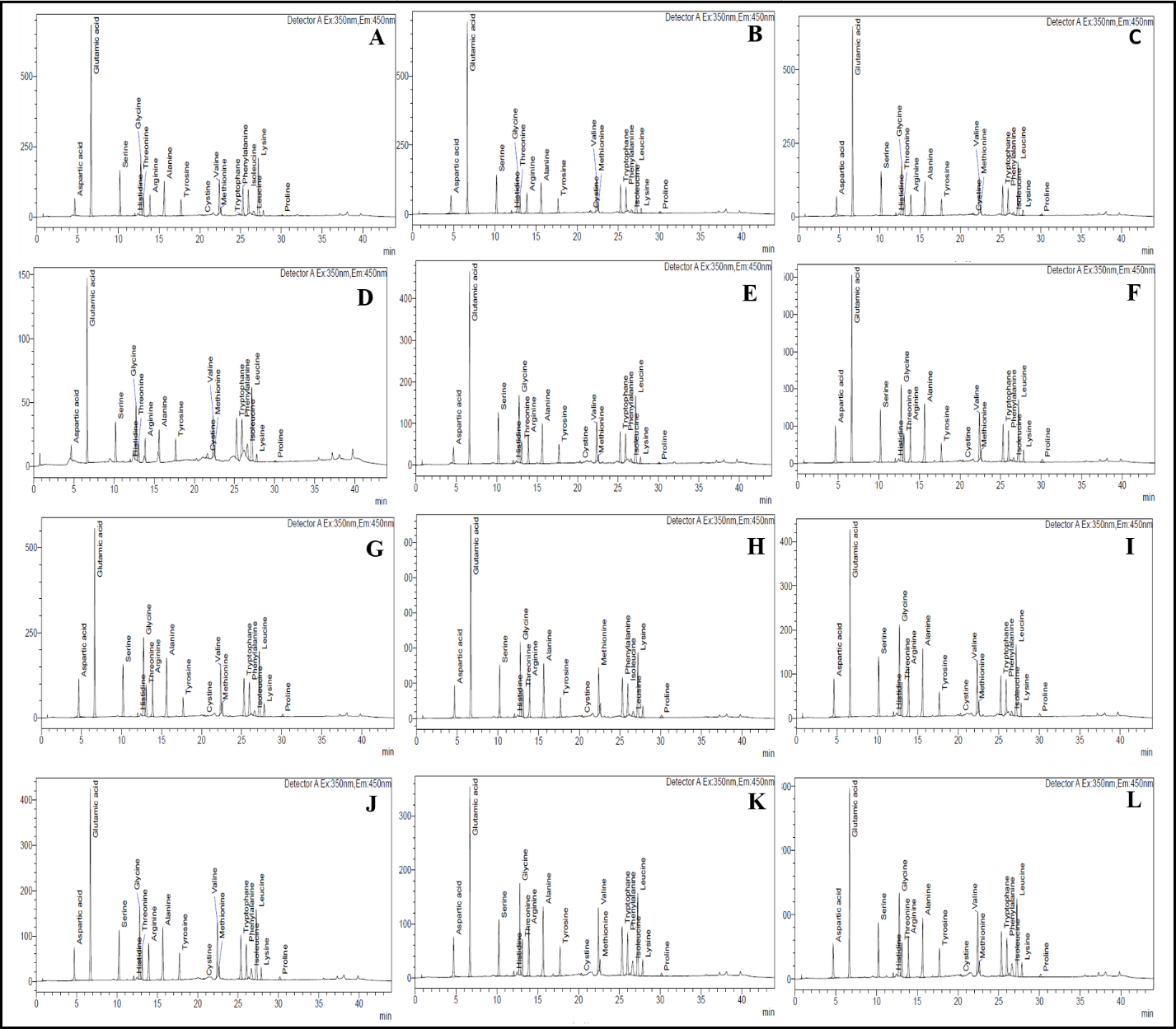


**Fig. S3.** HPLC Chromatograms displaying Amino acid profiling of wheat flour, A) HPW-147, B) HPW-155, C) HPW-236, D) HPW-249, E) HPW-349; rye flour: F) MCTLG-2, G) MCTLG-3, H) MCTLG-4, I) MCTLG-5 and Barley flour J) BH-902, K) BH-946, L) BH-959. Where X-axis and Y-axis exhibit the retention time in minute and intensity in mV respectively.
